# Protein profile of fiber types in human skeletal muscle: a single-fiber proteomics study

**DOI:** 10.1186/s13395-021-00279-0

**Published:** 2021-11-02

**Authors:** Marta Murgia, Leonardo Nogara, Martina Baraldo, Carlo Reggiani, Matthias Mann, Stefano Schiaffino

**Affiliations:** 1 Department of Biomedical Science, University of Padova, 35121 Padova, Italy; 2 Department of Proteomics and Signal Transduction, Max-Planck-Institute of Biochemistry, Martinsried, Germany; 3 Venetian Institute of Molecular Medicine (VIMM), 35121 Padova, Italy; 4 Science and Research Center Koper, Institute for Kinesiology Research, 6000 Koper, Slovenia; 5 NNF Center for Protein Research, Faculty of Health Sciences, University of Copenhagen, Copenhagen, Denmark

**Keywords:** Human skeletal muscle, Muscle fiber types, Single-fiber proteomics, Mass spectrometry

## Abstract

**Background:**

Human skeletal muscle is composed of three major fiber types, referred to as type 1, 2A, and 2X fibers. This heterogeneous cellular composition complicates the interpretation of studies based on whole skeletal muscle lysate. A single-fiber proteomics approach is required to obtain a fiber-type resolved quantitative information on skeletal muscle pathophysiology.

**Methods:**

Single fibers were dissected from vastus lateralis muscle biopsies of young adult males and processed for mass spectrometry-based single-fiber proteomics. We provide and analyze a resource dataset based on relatively pure fibers, containing at least 80% of either MYH7 (marker of slow type 1 fibers), MYH2 (marker of fast 2A fibers), or MYH1 (marker of fast 2X fibers).

**Results:**

In a dataset of more than 3800 proteins detected by single-fiber proteomics, we selected 404 proteins showing a statistically significant difference among fiber types. We identified numerous type 1 or 2X fiber type–specific protein markers, defined as proteins present at 3-fold or higher levels in these compared to other fiber types. In contrast, we could detect only two 2A-specific protein markers in addition to MYH2. We observed three other major patterns: proteins showing a differential distribution according to the sequence 1 > 2A > 2X or 2X > 2A > 1 and type 2–specific proteins expressed in 2A and 2X fibers at levels 3 times greater than in type 1 fibers. In addition to precisely quantifying known fiber type–specific protein patterns, our study revealed several novel features of fiber type specificity, including the selective enrichment of components of the dystrophin and integrin complexes, as well as microtubular proteins, in type 2X fibers. The fiber type–specific distribution of some selected proteins revealed by proteomics was validated by immunofluorescence analyses with specific antibodies.

**Conclusion:**

We here show that numerous muscle proteins, including proteins whose function is unknown, are selectively enriched in specific fiber types, pointing to potential implications in muscle pathophysiology. This reinforces the notion that single-fiber proteomics, together with recently developed approaches to single-cell proteomics, will be instrumental to explore and quantify muscle cell heterogeneity.

**Supplementary Information:**

The online version contains supplementary material available at 10.1186/s13395-021-00279-0.

## Background

Human skeletal muscles are composed of three major fiber types, slow type 1, fast 2A, and fast 2X fibers, defined by the presence of MYH7 (myosin heavy chain 7), MYH2, and MYH1, respectively, as well as hybrid fibers containing multiple MYHs, most frequently MYH7 + MYH2 (type 1–2A) or MYH2 + MYH1 (2A–2X) [1]. In contrast to other mammalian species containing muscles with predominant slow or fast fiber type profile, most human muscles are mixed in their fiber type composition. Enzyme and immuno-histochemistry on muscle sections or biochemical analyses on isolated single myofibers revealed a limited number of proteins differentially expressed in human fiber types. A major progress in this field has been possible with the introduction of single-fiber proteomics.

Muscle fibers are multinucleated single cells, whose predominant bulk mass consists of few highly abundant sarcomeric proteins. This limits the capability of a mass spectrometer to fragment and identify low-abundance protein species. In addition, isolated individual muscle fibers contain on average few micrograms of protein, hundreds of times less than typical starting amounts in proteomics just a few years ago. Recently, we set out to apply a highly sensitive mass spectrometry (MS)-based proteomic workflow to measure the proteome of the four fiber types (1, 2A, 2X, and 2B) present in mouse skeletal muscle [2]. We subsequently compared the proteomic profile of isolated human slow type 1 and fast 2A fibers from young and old individuals [3]. We did not analyze the fast 2X fibers in the latter comparative study, because pure type 2X fibers were not present in the older group.

The present study aims to compare the proteomic profile of type 2X fibers to that of type 1 and 2A fibers in young individuals. Our analysis contributes relevant information both on abundant muscle-specific proteins, whose distribution in different fiber types was not always known for human muscle, and on less-abundant less-characterized proteins, potentially relevant for muscle physiology and pathophysiology. Indeed, one major contribution of the single-fiber proteomics approach is its ability to detect muscle-intrinsic proteomic features, devoid of the contribution of non-muscle cells that contaminate the protein profile of whole muscle biopsies. We here provide a dataset with over four hundred proteins showing differential distribution in the three fiber types of human skeletal muscle. The results of the proteomic analyses were validated for selected proteins by immunofluorescence staining of muscle sections with specific antibodies.

## Methods

### Single-fiber proteomics

The proteomic analyses reported here are based on a previous study from our group, in which we measured the proteome of 152 single muscle fibers by liquid chromatography coupled to mass spectrometry (LC-MS) [3]. Data are deposited in ProteomeXchange with the accession number PXD006182. The fibers were isolated from muscle biopsies of four younger (aged 22–27 years) and four older (aged 65–75 years) healthy volunteers. Only the fibers isolated from the younger group are analyzed here. Experiments were performed with approval from the Ethics Committee of the University of Padua, Department of Biomedical Sciences (HEC-DSB08/16) (see [3]).

Biopsies were transferred to Petri dishes kept on ice immediately after surgery. Single fibers were manually dissected with tweezers, transferred to Eppendorf tubes and immediately frozen in liquid nitrogen within about 15 min after surgery. All sample processing and peptide purification steps were performed in a single vessel, thus minimizing sample loss. Liquid chromatography performed on an EASY-nLC 1200 ultra-high-pressure system on a 50-cm column of ReproSil-Pur C18-AQ 1.9-μm resin (Dr. Maisch) packed in house, coupled to a Q Exactive HF mass spectrometer (Thermo Fisher Scientific). A nonlinear 120-min gradient of 2–60% buffer B (0.1% [v/v] formic acid and 80% [v/v] acetonitrile) at a flow rate of 250 nL/min was applied. Data acquisition switched between a full scan and ten data-dependent MS/MS scans. Multiple sequencing of peptides was minimized by excluding the selected peptide candidates for 30 s [4]. The MaxQuant software (version 1.5.3.34) was used for the analysis of raw files, and peak lists were searched against the human UniProt FASTA reference proteomes version of 2016 and a common contaminants database by the Andromeda search engine [5, 6]. This identified more than 60,000 peptides and more than 5400 proteins, both at a false discovery rate (FDR) of 1%. Almost all proteins (92%) were quantified in all subjects. Bioinformatic and statistical analyses were performed with the Perseus software (version 1.5.4.2), part of the MaxQuant environment [7] using the MaxQuant label-free quantification (LFQ) algorithm [8].

For the present study, we selected relatively pure myofibers containing at least 80% of either MYH7 or MYH2 or MYH1. A total of 61 fibers (19 type 1, 29 type 2A, and 13 type 2X fibers) were thus examined. Given the high sequence identity of different MYHs, MYH expression was quantified by the intensities of peptides unique for each isoform [3]. To evaluate the relative abundance of each protein in the fiber proteome, the summed intensity of the peptides of each protein was divided by the number of theoretically observable peptides [8], and the resulting values were normalized to the expression of α-skeletal actin (ACTA1), as previously described [2, 3]. The complete dataset was filtered for at least 4 valid values in at least one fiber type group, which retrieved more than 3800 proteins (Dataset 1). The analysis of protein distribution among the 3 fiber types was based on the statistical comparisons with ANOVA, using 0.05 permutation-based false discovery rate (FDR) for truncation, followed by Tukey's HSD post hoc test. ANOVA significant proteins were the basis of all analyses. The ratios and percentage were calculated on the median values in each fiber type.

### Immunofluorescence

We examined biopsies of the vastus lateralis muscle from individuals (males, age range 18–35 years) participating in a study on the effect of eccentric vs. concentric resistance exercise [9]. The biopsies examined were obtained before the start of the exercise program. The study was approved by the University of Nottingham Ethics Committee and was performed in accordance with the Declaration of Helsinki (approval number B13032014 SoMSGEM). All participants provided written informed consent. We also used biopsies of the rectus abdominis muscle obtained in the context of a study on cancer cachexia from control patients without cancer (males, age range 40–74 years) undergoing surgery for diseases not affecting skeletal muscle [10]. All patients joined the protocol according to the guidelines of the Declaration of Helsinki and the research project was approved by the Ethical Committee for Clinical Experimentation of Provincia di Padova (protocol number 3674/AO/15). Dedicated written informed consent was obtained from participants. Immunostaining was carried out on completely anonymized residual samples.

Muscle 10 μm cryosections were stained with anti-MYH antibodies to reveal fiber types, using monoclonal antibodies BA-D5 and SC-71, which were originally described by Schiaffino et al. [11] and 6H1, originally described by Lucas et al. [12]. These three antibodies, which are distributed by Developmental Studies Hybridoma Bank (DSHB), were applied together using appropriate secondary antibodies, as described [3]. In addition, we used monoclonal antibody BF-35, also distributed by DSHB, which is reactive with MYH7 and MYH2 but not with MYH1, thus stains all fiber types, except pure type 2X fibers, both in the rat [11] and human muscles [1]. The following other antibodies were applied to serial sections: anti-PGM5/aciculin (Sigma, MABT1503-25UG), anti-PDLIM1 (DSHB, CPTC-PDLIM1), anti-MCU (D2Z3B, Cell Signaling Technology #14997), anti-IDH2 (Sigma, HPA007831), anti-ACTN3 (Boster Bio, PB10026), and anti-XIRP2 (Atlas Antibodies, HPA034813). Pretreatment with 0.1% Triton for 30 min for membrane permeabilization was used for mitochondrial proteins (MCU, IDH2). Secondary antibodies were AlexaFluor 594-conjugated IgG (Jackson ImmunoResearch: anti-rabbit, #111-585144, and anti-mouse, #115-585-146). Fiber profiles were revealed by Wheat Germ Agglutinin (WGA) staining (Thermo Fisher, W11261). Sections were examined with a Leica DM6B microscope, equipped with a DFC 7000T camera.

## Results

**Fig. 1:**
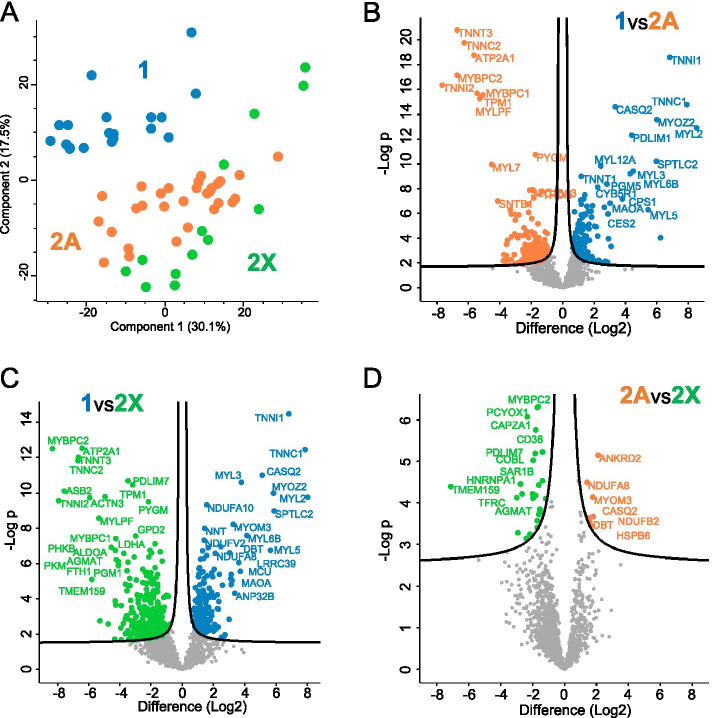
Global analysis of proteome differences between fiber types in human skeletal muscle, as determined by single-fiber proteomics. **A** Principal component analysis (PCA) performed on pure slow type 1 and fast 2A and 2X fibers (*n* = 61). Components were derived from proteins expressed in at least 5 fibers for each fiber type. MYHs were not considered. **B** Volcano plot of statistical significance against fold change, highlighting the most significantly different proteins between type 1 and 2A fibers. **C** Volcano plot as in **B** for type 1 vs 2X fibers. **D** Volcano plot as in **B** for type 2A vs 2X fibers

The expression values of all proteins detected in human fiber types are reported in Dataset 1. The expression values of each protein are normalized for the expression values of sarcomeric actin (ACTA1) to account for differences in fiber volume due to both fiber length and cross-sectional area. For each protein and fiber type, we provide the median expression values and the percent of the maximal value. The number of fibers in which each protein was identified (valid values) is also reported. Proteins are indicated by gene name. Principal component analysis (PCA) yielded a net diagonal separation into two groups, corresponding to type 1 (blue dots) and type 2 fibers (green and orange dots) along components 1 and 2. There was an overlap between the 2A and 2X fast subsets (Fig. 1A). Direct comparison of the proteome of different fiber types using volcano plots confirmed that there are many proteins with significant differential expression between type 1 and either 2A or 2X fibers, whereas the differences between 2A and 2X fibers are limited to a rather small number of proteins (Fig. 1B–D). Importantly, the few proteins marked as high in 2A fibers in Fig. 1D are actually not 2A specific, because they are expressed at high levels also in type 1 fibers (compare Dataset 1).

**Fig. 2:**
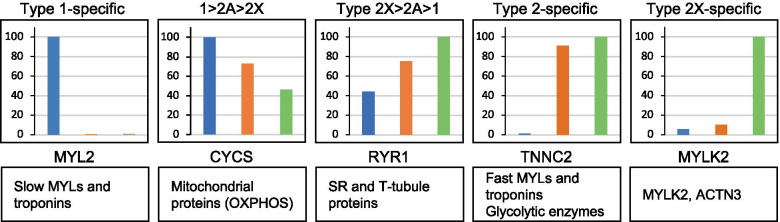
Major patterns of protein distribution according to fiber type in human skeletal muscle. The fiber type distribution of representative proteins (indicated by gene name) is shown for each pattern, with values expressed as per cent of the maximal value. *ACTN3*, α-actinin-3; *CYCS*, cytochrome c; *MYL2*, MLC-2 slow; *MYLK2*, myosin light chain kinase 2; *RYR1*, ryanodine receptor 1; *TNNC2*, fast troponin C

Some consistent fiber type–driven patterns emerged after a detailed analysis of individual protein distribution in the three fiber types. We focused on the expression analysis of 404 proteins showing a statistically significant difference between fiber types (ANOVA, *p* < 0.05) out of the dataset of more than 3800 proteins detected by single-fiber proteomics (Dataset 2 and see Methods). We first searched for proteins specifically expressed in either type 1 or 2A or 2X fibers. Our criterion for the identification of fiber type–specific markers was a threshold of at least 3-fold higher expression in one fiber type compared with the others. This way, we found 30 proteins specific for type 1 fibers and 41 proteins for 2X fibers, some of which are known to be functionally important in muscle physiology (Dataset 2 and Table 1). In contrast, surprisingly, only two 2A-specific proteins were identified with this procedure, corresponding to poorly characterized proteins (RDH11, a retinol dehydrogenase, and AIMP1, a component of the tRNA synthase complex) (Dataset 2 and Table 1). We subsequently screened the rest of this dataset according to the following 3 major patterns of protein expression: proteins expressed with at least 3-fold higher values in 2A and 2X compared with type 1 fibers and proteins showing a distribution 1 > 2A > 2X or 2X > 2A > 1, with a statistically significant difference between type 1 and 2X values. A large number of proteins displayed these 3 patterns of fiber type distribution, whereas only four proteins were expressed at 3-fold or higher values in type 1 and 2A compared with type 2X fibers (type 1–2A–specific pattern; Dataset 2 and Table 1). In subsequent analyses, we did not consider the type 2A–specific and the type 1–2A–specific patterns and focused on the 5 major patterns of fiber type distribution (Fig. 2), which account for 322 proteins (i. e., about 80% of the selected 404 proteins) (Table 1). The remaining 20% proteins (82 proteins) showed minor patterns of fiber type distribution, including a small number of proteins displaying an atypical pattern, with lower expression in 2A compared with 2X and type 1 fibers (1 > 2a, 2x > 2a) (Table S1). Notably, also in this group, only 3 proteins showed a type 2A–specific pattern, with values in 2A fibers significantly higher than in type 1 and 2X fibers, but less than 3 times higher.

**Table 1:** Muscle proteins showing a statistically significant difference between fiber types: major patterns^1^

*Pattern*	*Fiber type difference*^*2*^	*Threshold*	*No. of fibers*
Type 1-specific	1 > 2a; 1 > 2x	> 3 times	30
Type 2X-specific	2x > 1; 2x > 2a	> 3 times	41
Type 2A/2X-specific	2x > 1; 2a > 1	> 3 times	70
1 > 2A > 2X	1 > 2x	-	100
2X > 2A > 1	2x > 1	-	81
Total			322

^1^Minor patterns, shown by other 82 proteins, are shown in Table S1

^2^*P* < 0.05

A preliminary inspection of the composition of the selected 5 major protein patterns revealed that muscle contractile, sarcoplasmic reticulum (SR), and metabolic proteins fit easily into this scheme. Although their differential distribution in fast versus slow muscle is well-known, the specific fiber type distribution was not previously reported for human muscle in all cases (Fig. 2 and Table 2). Proteins showing the type 2X–specific profile are less characterized, with the exception of MYLK2 and ACTN3, and do not appear to belong to major pathways or structures of skeletal muscle. Examples of proteins showing a differential pattern of expression in the various fiber types are described below. We will consider proteins predominantly expressed in type 1/slow and type 2/fast fibers separately.

**Table 2:**
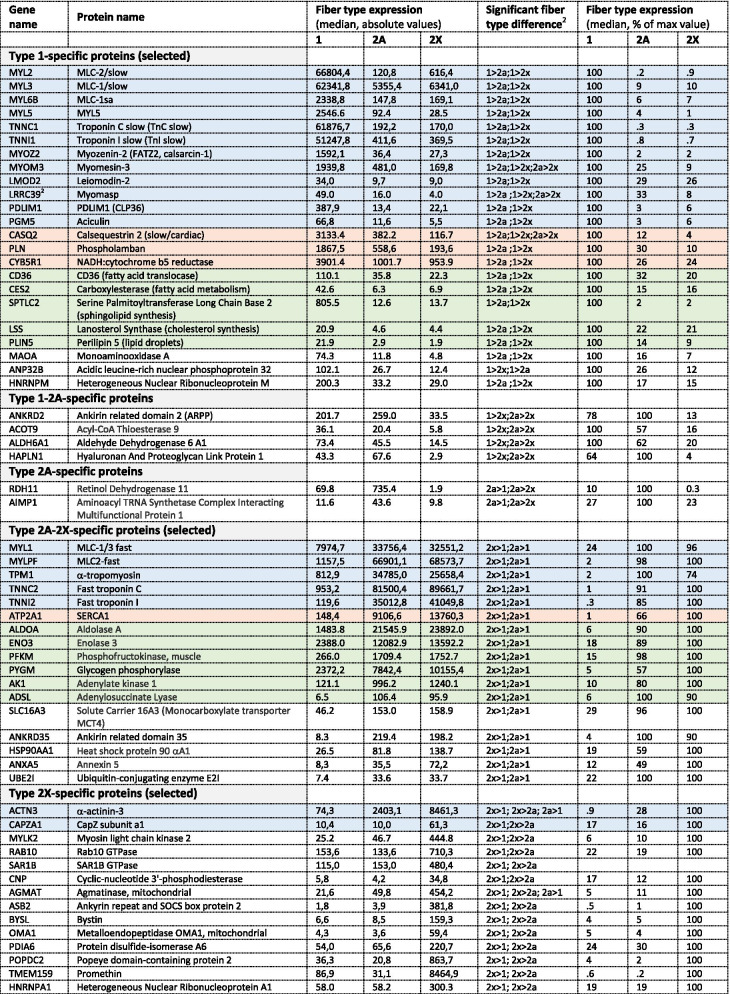
Proteins selectively expressed in specific fiber types in human skeletal muscle^1^

^1^Proteins whose level of expression is > 3 times higher in specific fiber types. All type 2A– and 1/2A–specific proteins are indicated, but only a selected list is presented for type1–, 2X–, and 2A/2x–specific protein. See Dataset 2 for a complete list. Color code: blue, contractile and cytoskeletal proteins; pink, SR proteins; green, metabolic pathways (lipid metabolism, glycolysis)

^2^*P* < 0,05

### Proteins enriched in type 1/slow fibers

**Fig. 3:**
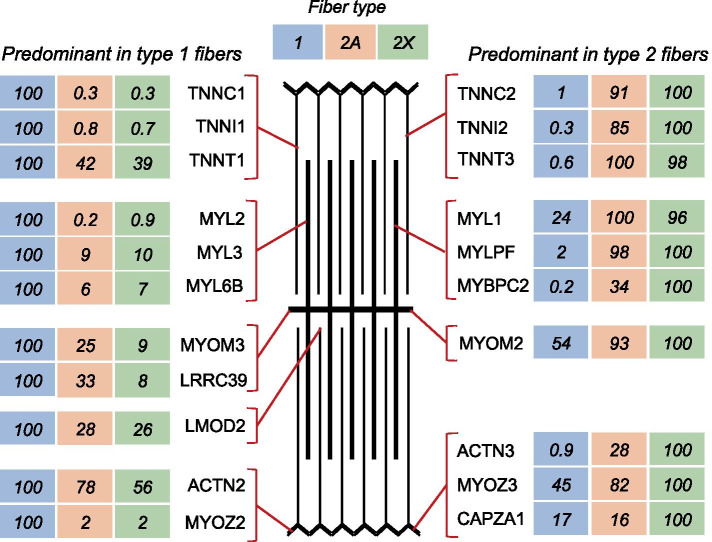
Myofibrillar proteins: fiber type distribution of representative proteins. Proteins showing similar distribution in the different types are not shown in the figure. Values are expressed as per cent of the maximal value. *ACTN2*, α-actinin-2; *ACTN3*, α-actinin-3; *CAPZA1*, CapZ α1; *LMOD2*, leiomodin-2; *LRRC39*, Myomasp; *MYL1*, MLC1/3-fast; *MYL2*, MLC-2slow; *MYL3*, MLC-1 slow; *MYLPF*, MLC2-fast; *MYL6B*, MLC-1sa; *MYBPC1*, myosin-binding protein C1; *MYBPC2*, myosin-binding protein C2; *MYBPH*, myosin-binding protein H; *MYOM2*, myomesin 2; *MYOM3*, myomesin 3; *MYOZ2*, myozenin 2; *MYOZ3*, myozenin 3; *TNNC1*, slow troponin C; *TNNC2*, fast troponin C; *TNNI1*, slow troponin I; *TNNI2*, fast troponin I; *TNNT1*, slow troponin T; *TNNT2*, fast troponin T; *TPM1*, α-tropomyosin; *TPM3*, γ-tropomyosin

The *type 1–specific pattern* is shown by numerous muscle proteins expressed with at least 3-fold higher values in type 1 compared with 2A and 2X fibers (Table 2 and Dataset 2). Along with MYH7, the main marker protein of this pattern, a most extreme form is represented by the slow myosin light chains, MYL3 (slow essential or alkali MYL, usually referred to as MLC-1/slow), and MYL2 (slow regulatory or phosphorylatable MYL, usually referred to as MLC-2/slow). The distribution of these and other myofibrillar proteins in human skeletal muscle is shown in Fig. 3, where the relative level of expression is indicated as percent of the maximum value, and in Table S2, which includes the absolute values and the statistical significance of the differences between fiber types. Fast 2A and 2X fibers contain only traces of MYL2, whereas MYL3 is present in significant amount also in the fast fibers, although it is 10 times less abundant in fast 2A and 2X compared with type 1 fibers. Slow type 1 fibers contain much lower, though significant amounts of two additional MYLs that are barely detectable in fast 2A and 2X fibers: MYL6B, an essential MYL selectively expressed in slow muscles, corresponding to a cDNA clone isolated from a human skeletal muscle library and referred to as MLC-1sa [13], and MYL5, the product of a ubiquitous regulatory MYL transcript reported to be expressed in fetal but not adult human muscle [14] and also present in diverse non-muscle tissues. The slow troponins, TNNC1 (slow troponin C) and TNNI1 (slow troponin I), are also hundreds of times more abundant in type 1 compared with type 2 fibers. In contrast, TNNT1 (slow troponin T) is present in high amount in all fiber types, being only 2 times more abundant in slow compared with fast fibers. Other myofibrillar proteins, such as MYOZ2 (myozenin-2 *alias* FATZ2 *alias* calsarcin-1), MYOM3 (myomesin-3), and LMOD2 (leiomodin-2) display this pattern of fiber type distribution. The type 1–specific pattern is detectable not only in myofibrillar proteins but also in some components of the sarcoplasmic reticulum (SR), including calsequestrin 2 (CASQ2), a well-known marker of cardiac and slow skeletal muscle, PLN (phospholamban), a small protein associated to the slow SERCA2 Ca^2+^ pump of the SR, and CYB5R1 (NADH-cytochrome b5 reductase), a protein of unknown function that was previously reported to be enriched in microsomal membranes of rabbit slow skeletal muscle [15].

Most of these results are essentially in agreement with previous studies in different mammalian species. However, those findings were not always validated in human muscle and mostly not examined at the single fiber level. Our proteomic approach revealed the existence of novel type 1–specific proteins, whose fiber type distribution has not been previously reported. These include LRRC39 (Myomasp), a leucine-rich sarcomeric protein bound to myosin and located at the M-band [16], PDLIM1 (*alias* CPL36), a member of the α-actinin–associated LIM protein (ALP) subfamily of the PDZ-LIM family of proteins [17], and PGM5 (aciculin), a protein associated with the actin cytoskeleton and interacting with filamin C and Xin [18], which is especially abundant at the myotendinous junctions and costameres (Table S3). Other type 1–specific proteins are involved in lipid metabolism, such as LSS (lanosterol synthase), a key enzyme in the cholesterol biosynthesis pathway, SPTLC2, a subunit of serine palmitoyltransferase, the enzyme responsible for the first step in sphingolipid synthesis [19], and CD36 (fatty acid translocase), the predominant plasma membrane protein responsible for fatty acid uptake [20]. A similar fiber type distribution is shown by some mitochondrial proteins, such as MAOA (monoaminoxidase A) and MCU (mitocondrial Ca^2+^ uniporter). Another example of type 1–specific protein is ANP32B, which has been recently reported to be significantly increased in the serum of Duchenne muscular dystrophy patients [21]. ANP32B is a member of the acidic nuclear phosphoprotein 32kDa (ANP32) family, composed of protein isoforms with diverse and often opposed biochemical activities [22]. Indeed, another isoform present in muscle, ANP32A, has an opposite expression pattern, being a highly enriched in type 2X fibers. The divergent fiber type distribution of members of the same protein family is also shown by heterogeneous nuclear ribonucleoproteins (hnRNPs), a family of proteins known to be involved in alternative splicing and different aspects of RNA metabolism [23]: HNRNPM is selectively enriched in type 1 fibers, whereas HNRNPD and HNRNPA1 are type 2X–specific proteins (Table 2 and Dataset 2).

**Fig. 4:**
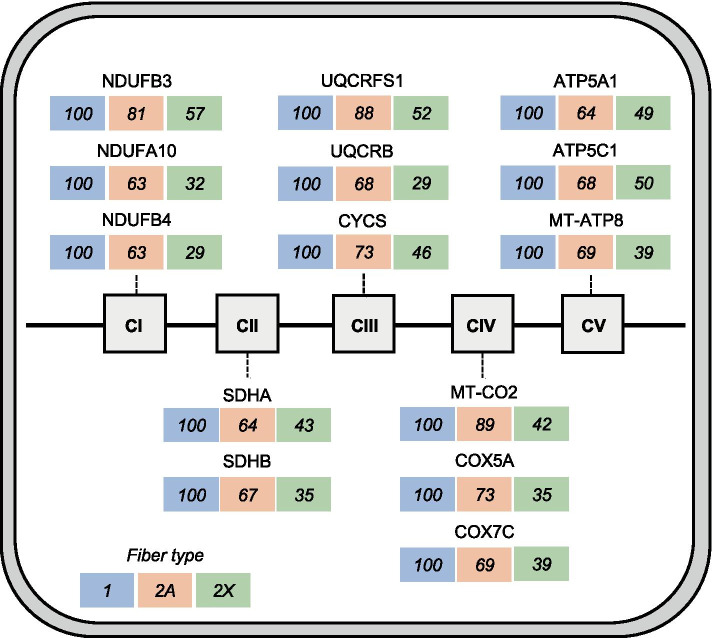
Mitochondrial proteins: fiber type distribution of representative OXPHOS proteins. Representative subunits of the electron transport chain complexes (CI to CIV) and of the ATP synthase complex (CV) are shown. *CI*, NADH:ubiquinone oxidoreductase; *CII*, succinate dehydrogenase; *CIII*, ubiquinol-cytochrome C reductase; *CIV*, cytochrome C oxidase; *CV*, ATP synthase. Values are expressed as per cent of the maximal value

**Fig. 5:**
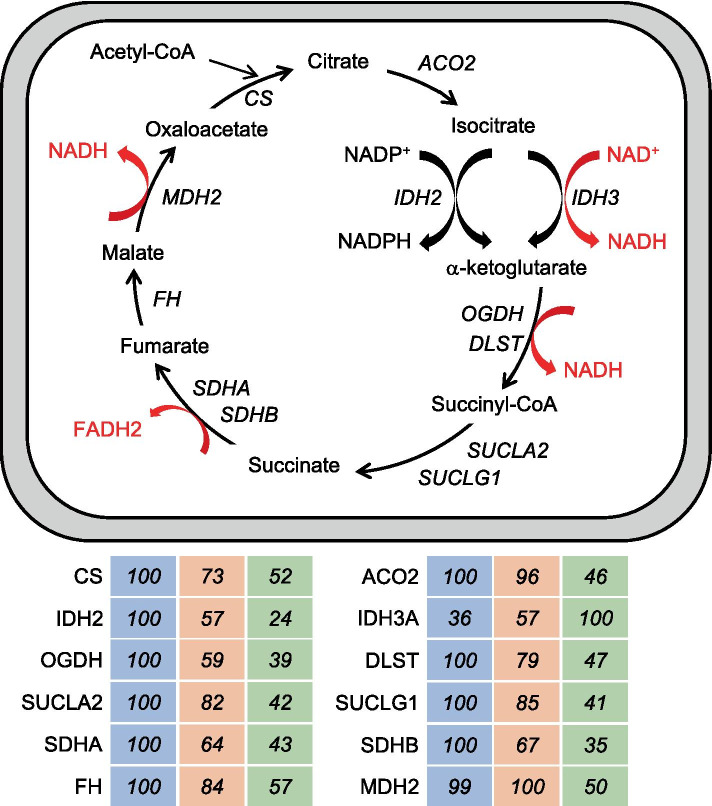
Mitochondrial proteins: fiber type distribution of TCA cycle proteins. A scheme of the TCA cycle is shown in the upper panel and the fiber type distribution of the constituent enzymes in the lower panel. *ACO2*, aconitase; *CS*, citrate synthase; *DLST*, dihydrolipoamide S-succinyltransferase; *FH*, fumarate hydratase; *IDH2*, isocitrate dehydrogenase, NADPH-dependent; *IDH3A*, isocitrate dehydrogenase, NADH-dependent, subunit α; *MDH2*, malate dehydrogenase, mitochondrial; *OGDH*, oxoglutarate dehydrogenase; *SDHA*, succinate dehydrogenase subunit A; *SDHB*, succinate dehydrogenase subunit B; *SUCLA2*, succinate-CoA ligase ADP-forming subunit β; *SUCLG1*, succinate-CoA ligase GDP/ADP-forming subunit α

The *type 1 > 2A > 2X* pattern defines proteins showing highest values in type 1, intermediate values in 2A, and lowest values in 2X fibers, with a statistically significant difference between type 1 and 2X values. This is the most frequent pattern shown by mitochondrial proteins, such as electron transport chain and oxidative phosphorylation (OXPHOS) proteins (Fig. 4). Considering all OXPHOS proteins from Complex I to Complex V present in our database, the following average percent values were obtained in the three fiber types: type 1 fibers, 100; 2A fibers, 77; 2X fibers, 46. Most other mitochondrial proteins are, likewise, 2–3 times more abundant in type 1 than 2X fibers, with intermediate values in 2A fibers. This is true for proteins localized in the mitochondrial matrix, like TCA cycle enzymes (Fig. 5) and fatty acid β-oxidation enzymes, the inner membrane, like the ATP/ADP carrier (SLC25A4), the mitochondrial intermembrane space, like mitochondrial creatine kinase (CKMT2), and the outer membrane, like the mitochondrial voltage-dependent anion channel (VDAC1). However, a number of mitochondrial proteins have a different pattern of fiber type distribution. For example, while the mitochondrial calcium uniporter (MCU) is more than tenfold enriched in type 1 compared with 2X fibers (see above), OPA1, involved in mitochondrial fusion, shows no significant difference in fiber type distribution, and isocitrate dehydrogenase 3α (IDH3A) is expressed at higher levels in 2X than type 1 fibers (Fig. 5), in agreement with previous proteomic observations on mouse single-muscle fibers [3, 24]. The mitochondrial components of the glycerol–phosphate and malate–aspartate shuttles also have an atypical fiber type distribution (see below). The 1 > 2A > 2X pattern is also shown by the intermediate filament protein DES (desmin), by the myofibrillar proteins MYBPC1 (myosin-binding protein C1) and MYOM3 (myomesin-3) (Tables S2 and S3) and a large number of other muscle proteins (Dataset 2).

### Proteins enriched in type 2/fast fibers

**Fig. 6:**
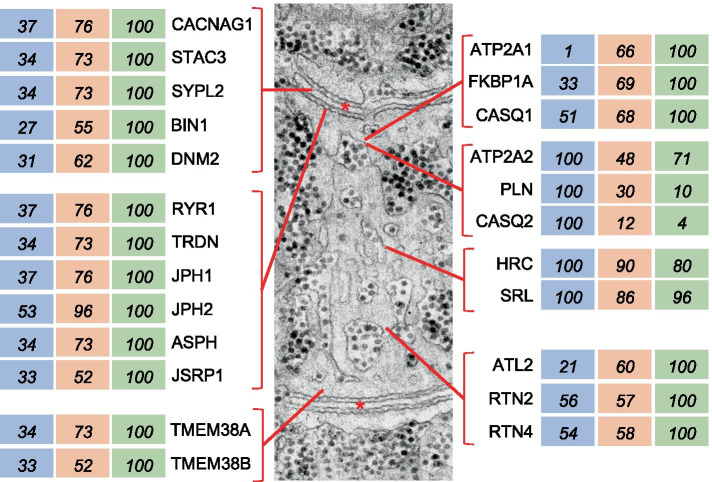
T tubule and SR proteins: fiber type distribution of representative proteins. The electron micrograph in the central panel shows a face view of the SR, with T tubules labeled by asterisks. Values are expressed as per cent of the maximal value. *ASPH*, junctin/junctate; *ATL2*, atlastin 2; *ATP2A1*, SERCA 1 (sarco(endo)plasmic reticulum calcium-ATPase 1); *ATP2A2*, SERCA 2; *BIN1*, bridging integrator 1 (amphiphysin 2); *CACNAG1*, voltage-dependent calcium channel Ca_V_1.1 (dihydropyridine receptor, DHPR) γ1 subunit; *CASQ1*, calsequestrin 1; *CASQ2*, calsequestrin 2; *DNM2*, dynamin 2; *FKBP1A*, FK binding protein 1A, prolyl isomerase 1A; *HRC*, histidine rich Ca^2+^ binding protein; *JPH1*, junctophilin 1; *JPH2*, junctophilin 2; *JSRP1*, JP-45; *PLN*, phospholamban; *RTN2*, reticulon 2; *RTN4*, reticulon 4; *RYR1*, ryanodine receptor 1; *SRL*, sarcalumenin; *STAC3*, SH3 and cysteine-rich domain-containing protein 3; *SYPL2*, Mg29 (mitsugumin 29); *TMEM38A*, TRIC-A (trimeric intracellular cation channel A); *TMEM38B*, TRIC-B; *TRDN*, triadin (EM picture: modified from ref [28])

The *type 2X > 2A > 1 pattern* is typical of most SR and T tubule proteins, which display highest values in type 2X, intermediate values in 2A, and lowest values in type 1 fibers, with a statistically significant difference between type 1 and 2X values (Fig. 6 and Table S4). This is the case of the subunits of the voltage-dependent calcium channel Ca_v_1.1, also called dihydropyridine receptor, and other T tubule proteins, including STAC3 (SH3 and cysteine-rich protein 3), SYPL2 (synaptophysin-like protein 2, MG29), as well as two proteins involved in T tubule formation and membrane remodeling, amphiphysin 2 (BIN1) and dynamin 2 (DNM2) [25, 26]. The same pattern of fiber type distribution is typical of SR proteins involved in Ca^2+^ homeostasis, the Ca^2+^ release channel RYR1 (ryanodine receptor 1) and calsequestrin 1 (CASQ1), as well as the proteins responsible for the physical connection between SR and T tubules, triadin (TRDN), junctin/junctate (ASPH), and the junctophilins JPH1 and JPH2. Proteins involved in ER/SR shaping and branching, like atlastin 2 (ATL2), the reticulons RTN2 and RTN4, and REEP5 (DP1) [27] are also enriched in type 2X fibers, whereas two proteins located in the SR lumen, sarcalumenin (SRL) and histidine-rich Ca^2+^-binding protein (HRC), do not display fiber type–specific differences. The same pattern is also shown by proteins involved in Ca^2+^ homeostasis, like calmodulin and many of its targets, including SR and T tubule components (Fig. S1). However, other calmodulin targets, such as CAMKII subunits (CAMK2A, CAMK2B, CAMK2D, CAMK2G) do not show significant variation in the expression in different fiber types (Dataset 1). A progressively decreasing level of expression from 2X to 2A to type 1 fibers is shown by the myofibrillar proteins MYOZ3 (myozenin-3) and MYBPH (myosin-binding protein H), and by PDLIM7 (PDZ and LIM domain protein 7, *alias* Enigma), a PDZ-LIM protein highly expressed in 2X fibers (Tables S2 and S3).

Interestingly, the list of proteins enriched in type 2X fibers includes different components of the dystrophin complex, including SGCD (sarcoglycan γ), SNTB1 (β1-syntrophin), and SSPN (sarcospan), as well as ITGB1 (integrin β1), which together with ITGA7 (integrin α7) is the major integrin in muscle cells, and by proteins involved in membrane repair, such as DYSF (dysferlin) and ANXA5 (annexin 5) (Table S5). Most other components of the dystrophin and integrin complexes, and of membrane repair systems, show a similar pattern of fiber type distribution, although the difference between type 1 and 2X values was not statistically significant for many of them (Table S5). Another protein involved in membrane repair, TRIM72 (MG53) is expressed at similar levels in different fiber types; however, PTRF (CAVIN-1), which acts as an essential docking protein for MG53 during membrane repair [29], is more abundant in type 2X than type 1 fibers. LGALS1 (galectin-1), a glycan-binding protein that was found to improve sarcolemma stability in dystrophin-deficient muscles [30] and membrane repair in dysferlin-deficient models [31], is about 5 times more abundant in 2X compared with that in type 1 fibers (Table S5).

Many microtubule and microtubule-associated proteins display this pattern of fiber type distribution (Table S3). This is the case of the most abundant tubulin subunits, TUBA4A (Tubulin α-4A) and TUBA1B (Tubulin α-1B), as well as minor tubulin isoforms and microtubule-associated proteins, such as MAP1LC3B, usually referred to as LC3. LC3 is better known for its central role in autophagy but was recently shown to affect microtubule stability in the axons [32]. Many subunits of kinesins and dyneins, the motor proteins associated to microtubules, also tend to be enriched in type 2X compared with those in type 1 fibers, and the difference is statistically significant for dynactin 1 and 2 (DCTN1 and DCYN2), components of a microtubule-binding multiprotein complex involved in the recruitment of dyneins and their cargos onto microtubules [33] (Dataset 2).

**Fig. 7:**
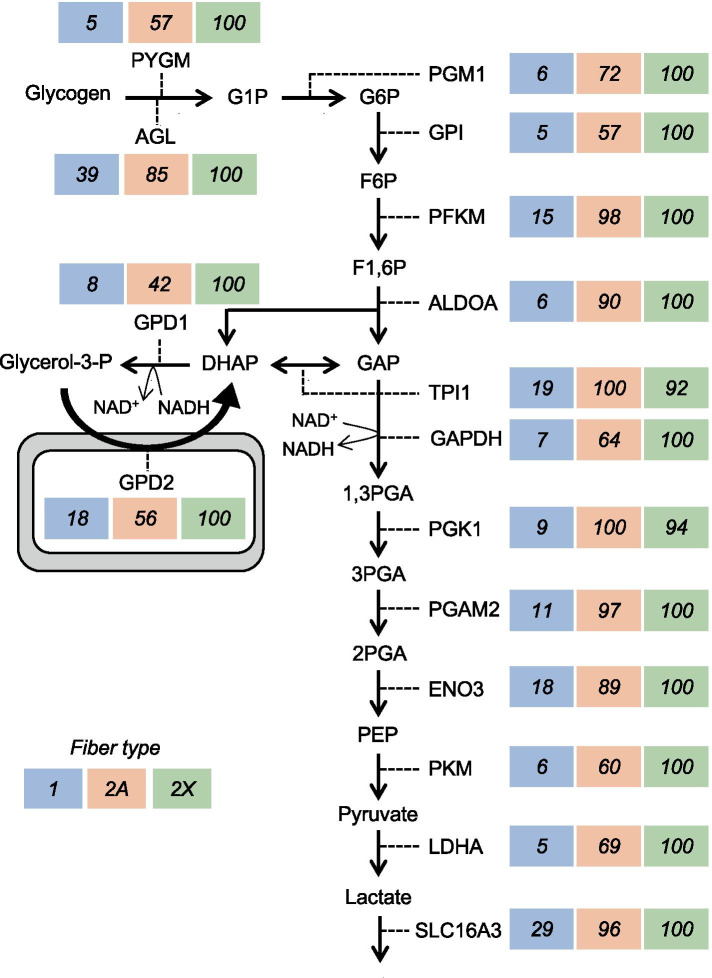
Glycolysis, glycogenolysis, and glycerol phosphate shuttle: fiber type distribution of representative proteins. Values are expressed as per cent of the maximal value. *AGL*, debranching enzyme; *ALDOA*, aldolase; *ENO3*, enolase 3 (β-enolase); *GAPDH*, glyceraldehyde 3-phosphate dehydrogenase; *GP1*, glucose-6-phosphate isomerase; *GPD1*, glycerol-3-phosphate dehydrogenase 1 (cytosolic); *GPD2*, glycerol-3-phosphate dehydrogenase 2 (mitochondrial); *LDHA*, lactate dehydrogenase A; *PFKM*, phosphofructokinase, muscle isoform; *PGAM2*, phosphoglycerate mutase 2; *PGK1*, phosphoglycerate kinase 1; *PGM1*, phosphoglucomutase 1; *PKM*, pyruvate kinase, muscle isoform; *PYGM*, glycogen phosphorylase; *TPI1*, triosephosphate isomerase 1

A type 2X > 2A > 1 pattern of fiber type distribution is also shown by the enzymes of the glycerol phosphate shuttle, including cytosolic GPD1 (glycerol-3-phosphate dehydrogenase 1) and mitochondrial GPD2 (glycerol-3-phosphate dehydrogenase 2), involved in the oxidation of NADH generated during the glycolytic pathway (Fig. 7 and Table S6). GPD1 uses the NADH generated by glyceraldehyde 3-phosphate dehydrogenase (GAPDH) to reduce dihydroxyacetone phosphate (DHAP) to glycerol 3-phosphate, which is oxidized back to DHAP by GPD2 with concomitant reduction of the flavin adenine dinucleotide and electron flow through the electron transport chain [34]. A different pattern of fiber type distribution is shown by the components of the malate–aspartate shuttle, which is also involved in the oxidation of glycolytic NADH [35]. As illustrated in Fig. S2 and Table S6, all six components of this shuttle, including the cytosolic and mitochondrial malate dehydrogenase (MDH1 and MDH2) and aspartate aminotransferase (GOT1 and GOT2), as well as the mitochondrial carriers SLC25A11 (oxoglutarate–malate carrier) and SLC25A12 (aspartate–glutamate carrier, also called aralar), were most abundant in type 2A fibers. However, the difference between 2A and 2X fibers was never more than 2 times, and that between 2A and type 1 fibers was quite variable, with the cytosolic components showing much lower levels in type 1 fibers and the mitochondrial components being essentially identical in the two fiber types. Interestingly, the other aspartate–glutamate carrier (SLC25A13, also called citrin), which is especially abundant in liver, shows the same tendency for highest levels in type 2A fibers, although its expression is much lower than that of SLC25A12 (Table S6). A previous study on isolated human muscle fibers reported that while GPD1 activity is higher in type 2 fibers, the four enzymes of the malate–aspartate shuttle show higher activity in type 1 fibers [36]; however, the type 2 fiber subgroups were not considered in this study.

The *type 2–specific* pattern is typical of proteins which are at least 3 times more abundant in type 2A and 2X compared with type 1 fibers. This pattern is shown by many myofibrillar proteins, like the fast myosin light chains MYL1 (fast essential or alkali MYL, usually referred to as MLC1/3-fast) and MYLPF (fast regulatory MYL, usually referred to as MLC2-fast), the fast troponins TNNC2 (fast troponin C), TNNI2 (fast troponin I) and TNNT2 (fast troponin T), and TPM1 (α-tropomyosin) (Fig. 3 and Table S2). All these proteins are hundreds of times more abundant in fast type 2 compared with slow type 1 fibers. Fast type 2 fibers also contain lower, though significant amounts of MYL7, a regulatory MYL that is barely detectable in slow type 1 fibers but is known to be abundant in the atrial myocardium. ATP2A1 (SERCA1), the calcium pump predominant in the SR of fast muscle is likewise present at 50–100 higher levels in 2A and 2X than in slow fibers (Fig. 6 and Table S4).

A type 2–specific pattern is shown by glycolytic enzymes, many of which showed a tendency for slightly lower levels in 2A compared with 2X fibers (Fig. 7 and Table S6). A prevalent distribution in type 2 fibers, according to a type 2–specific or 2X > 2A > 1 pattern, is found with glycogen phosphorylase (PYGM), the three subunits (α1, β, and γ1) of glycogen phosphorylase kinase (PHKA1, PHKB, and PHKG1) (Fig. S1), and with AK1 (adenylate kinase), involved in ATP buffering, as well as AMPD1 (AMP deaminase) and the other enzymes of the purine nucleotide cycle (Fig. S3). The removal of AMP to IMP by AMP deaminase is essential to avoid the accumulation of ATP hydrolysis products and thus protect the energy state of the muscle fibers during the contractile activity of fast-twitch muscle fibers, with the subsequent two reactions of the purine nucleotide cycle mediating the reamination of IMP and reconstitution of AMP [37, 38]. The monocarboxylate transporter MCT4, coded by SLC16A3, which is involved in exporting lactic acid from skeletal muscle [39], is also about 3 times more abundant in 2A and 2X than in type 1 fibers (Table 2). Among the proteins showing this pattern, one finds several other proteins not previously analyzed with respect to fiber type, including PRKAR1A (cAMP-dependent protein kinase regulatory subunit 1α), a component of protein kinase A (PKA), the kinase mediating the effect of β-adrenergic stimulation in muscle cells, and UNC45B, a myosin chaperone whose mutation causes a congenital myopathy [40, 41]. The same is true for ANRD35 (ankyrin repeat domain 35), a protein whose function is completely unknown: the fiber type distribution of ANRD35 is specular to that of ANKRD2, which is one of the rare proteins selectively enriched in type 1 and 2A fibers (Tables 2 and S3). ANKRD2, a member of the muscle ankyrin repeat protein (MARP) family of proteins linked to muscle cytoskeleton, is responsive to mechanical stretch [42] and was previously reported to be present in slow fibers of human skeletal muscle [43]. A similar situation, with two isoforms showing an opposite pattern of fiber type distribution, is that of XIRP1 (Xin), predominant in type 1 fibers, and XIRP2 (Xin2, beta-Xin), predominant in type 2X fibers (Table S3).

The *type 2X–specific* includes proteins showing the highest expression levels in type 2X fibers, with at least 3 times higher values compared with 2A and type 1 fibers (Table 2). A number of muscle proteins met these criteria, including myofibrillar proteins, such as ACTN3 (α-actinin-3), known to be enriched in fast glycolytic muscle fibers in different mammalian species [44], and CAPZA1 (CapZ, capping actin protein of muscle Z-line, subunit α1) (Fig. 3). A type 2X–specific pattern is also shown by MYLK2 (myosin light chain kinase 2, MLCK2), a Ca^2+^–calmodulin–dependent kinase that was known to be more abundant in fast-twitch muscles [45], although the specific fiber type was not previously identified. Other proteins selectively enriched in type 2X fibers are RAB10, a Rab GTPase known to regulate ER dynamics and morphology [46], CNP (2’,3’-cyclic-nucleotide 3’-phosphodiesterase), a microtubule-associated protein that may act as a linker protein between microtubules and the plasma membranes [47], and other muscle proteins whose function has been poorly characterized, such as ASB2, an E3 ubiquitin ligase, SAR1B, a small GTPase abundant in skeletal muscle, and AGMAT (agmatinase), an enzyme involved in polyamine metabolism (Table 2).

### Immunofluorescence analyses

**Fig. 8:**
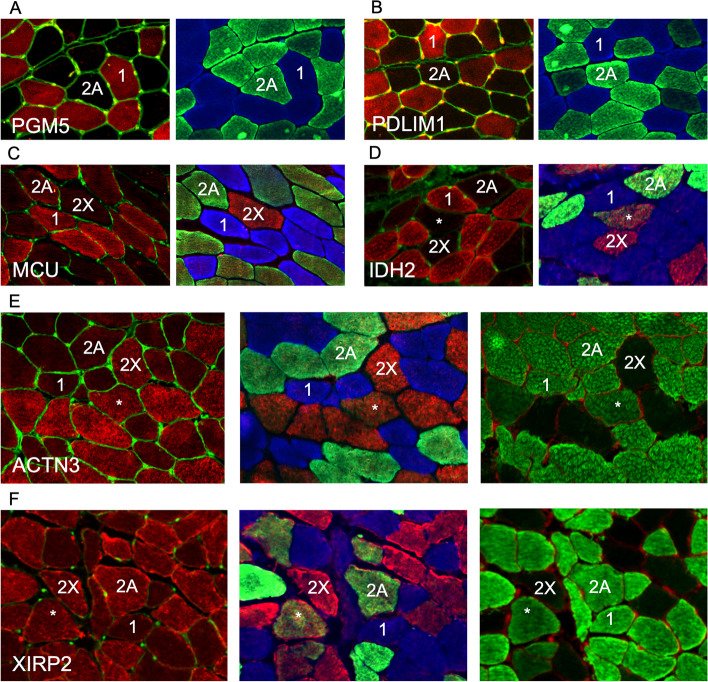
Fiber type–specific distribution of selected proteins revealed by immunofluorescence analysis. Fiber types are labeled as 1 (type 1), 2A (type 2A), or 2X (type 2X). Hybrid 2A–2X fibers are labeled by asterisks. **A**–**D** Type 1–specific proteins. Each panel shows on the left a section stained for PGM5/aciculin (**A**), PDLIM1 (**B**), MCU (**C**), or IDH2 (**D**) and on the right a serial section stained with MYH-specific antibodies to reveal the three fiber types. No pure type 2X fibers is present in **A** and **B**. **E**–**F** Type 2–specific proteins. Each panel shows on the left a section stained for ACTN3 (**E**) or XIRP2 (**F**), on the center a serial section stained for MYH-specific antibodies to reveal the three fiber types, and on the right a section stained with anti-MYH antibody BF-35, which reacts with MYH7 and MYH2 but not with MYH1, thus stains all fiber types, except pure type 2X fibers. Note that whereas ACTN3 is especially abundant in type 2X fibers, as well as in hybrid 2A–2X fibers, XIRP2 is present at higher levels in both 2A and 2X fibers. WGA counterstain was applied to all sections, except those processed with the three anti-MYHs antibodies

To validate the single-fiber proteomics data using an independent approach, we performed immunofluorescence analyses for some selected proteins, whose fiber type distribution in human skeletal muscle has not been previously investigated. As shown in Fig. 8A–D, we confirmed that PDLIM1, PGM5 (aciculin), MCU (mitochondrial Ca^2+^uniporter), and IDH2 (isocitrate dehydrogenase 2) are specifically expressed in type 1 fibers. Interestingly, the clear-cut difference between type 1 and both 2A and 2X staining shown by anti-MCU and anti-IDH2 is not seen with antibodies to traditional mitochondrial markers, such as TOM20 or OXPHOS proteins, which show a slightly more intense staining in type 1 compared with 2X, but no obvious difference between 1 and 2A (not shown). Accordingly, succinate dehydrogenase enzyme histochemistry shows weak differences among human fiber types, especially between type 1 and 2A fibers [48]. This result is consistent with the proteomic data showing that most mitochondrial proteins are only slightly more abundant in type 1 compared with 2A fibers, whereas MCU is enriched about 3-fold in type 1 compared with 2A fibers and even more compared with 2X fibers (see Figs. 4 and 5, and Datasets 1 and 2). Figure 8E and F illustrate immunofluorescence staining for type 2–specific proteins: ACTN3 shows a clear type 2X–specific pattern of expression, whereas XIRP2 is present at higher levels in type 2 compared with type 1 fibers, without detectable differences between type 2 fiber subpopulations. These staining patterns are consistent with proteomic data, with ACTN3 showing a greater difference between type 1 and 2A fibers compared with XIRP2 (Fig. 3 and Tables S2 and S3). However, immunohistochemistry cannot provide quantitative data about the relative proportion of a given protein in different fiber types.

## Discussion

The results of the present study provide a global picture of the protein composition of human type 1, 2A, and 2X fibers. Here, we extend the established knowledge based on previous studies from different mammalian species, by quantifying thousands of proteins from single muscle fibers and focusing on over four hundred proteins with statistically significant distribution in different fiber types. Among the proteins with differential expression, we measured major contractile proteins, including many troponins and myosin light chains. We confirmed the prevalence of mitochondrial oxidative enzymes in type 1 and of glycolytic enzymes in type 2 fibers. These findings are a proof of concept that single-fiber proteomics provides quantitative data which faithfully match established knowledge on skeletal muscle structure and function. Based on this solid ground, we here provide, in addition, novel quantitative information about protein components of muscle structures and metabolic pathways not previously linked to a specific fiber type. The results of the proteomic analyses were confirmed for selected proteins using an independent approach, namely immunofluorescence staining of muscle sections with specific antibodies.

A major result of this study concerns the very notion of fiber types in human skeletal muscle. Fiber types have been traditionally distinguished by the type of MYH they express, thus human fibers are classified as type 1, 2A, and 2X fibers based on the presence of MYH7, MYH2, or MYH1, respectively [1]. The first question we asked is whether human muscle fibers can be distinguished by other fiber type–specific protein markers, in addition to MYHs. To identify these markers, we have screened our database, composed of more than 3800 proteins, looking for proteins at least 3-fold more abundant in a given fiber type than in other fiber types. Many type 1 markers were retrieved by this analysis, including slow isoforms of myosin light chains, troponin C, and troponin I, which are more than 100-fold enriched in type 1 compared with 2A and 2X fibers. Likewise, a large number of markers specific for type 2 fibers and common to both type 2A and 2X fibers were also highlighted with this method, including fast-type isoforms of myosin light chains and troponins as well as glycolytic and other enzymes committed to the rapid provision of ATP required for fast muscle contraction. In contrast, type 2A and 2X fibers were not easily distinguished by these criteria, nor by global quantitative comparisons (Fig. 1). In particular, single-fiber proteomics did not reveal any type 2A–specific marker, except for MYH2 and few proteins whose significance is not clear. It should be stressed that our dataset contains other proteins which are expressed at a higher level in type 2A compared with those in type 1 and 2X fibers; however, these differences did not reach statistical significance. For example, as shown in Table S3, this pattern of fiber type distribution is shown by IGFN1, a protein localized at the Z-disc and interacting with KY, FLNC, and COBL [49, 50]. On the other hand, type 2X fibers are characterized by a significant number of 2X-specific proteins, as well as by the highest levels of most SR and T tubule proteins, reflecting the greater development of these membrane systems in type 2X–2B fibers in different species [51, 52].

The picture that emerges from these findings shows that human skeletal muscles consist essentially of two major fiber types, slow/type 1 and fast/type 2, with relatively minor differences between the type 2A and 2X subsets. The identity of 2A fibers with respect to 2X fibers, in addition to the unique MYH2 expression, is essentially defined by (i) higher levels of mitochondrial proteins, (ii) lower levels of proteins involved in E–C coupling, and (iii) very low levels of a limited number of 2X-specific proteins. An interesting difference between 2A and 2X fibers concerns the shuttle systems used for the oxidation of cytosolic NADH by mitochondria: the components of the glycerol–phosphate shuttle, GPD1 and GPD2, are more abundant in 2X fibers, whereas the components of the malate–aspartate shuttle tend to show higher values in 2A fibers. The interpretation of the level of a given enzyme in a metabolic pathway is complicated by the multiple roles of some of these enzymes in different pathways: for example, the high levels of MDH2 in both type 1 and 2A fibers could reflect the fact that this enzyme is both a component of the TCA cycle, characterized by higher levels in type 1 fibers, and of the malate-aspartate shuttle, characterized by higher levels in type 2A fibers.

A specific feature of human type 2X fibers revealed by single-fiber proteomics is the presence of high levels of MYLK2, which is about 10-fold more abundant than in 2A and even more in type 1 fibers. The high levels of MYLK2 in 2X fibers appear to be functionally relevant because phosphorylation of the regulatory myosin light chain by MYLK2 leads to an increase in the rate of force generation by myosin crossbridges and potentiation of isometric force in intact muscle [45]. In particular, studies on permeabilized muscle fibers have shown that myosin phosphorylation increases the force output and the rate of force development at low levels of Ca^2+^ activation, thus increasing the Ca^2+^ sensitivity of the contractile machinery [45]. In humans, the effect of myosin phosphorylation is revealed by the so-called post-activation (or post-tetanic) potentiation, namely the enhanced twitch contraction after a brief maximal contraction: this effect was found to correlate with the total cross-sectional area of type 2 fibers; however, the specific role of fast fiber subtypes in post-activation potentiation and myosin light chain phosphorylation was never determined in previous studies [53]. MYLK2 activity is controlled by Ca^2+^–calmodulin, and our results show that both calmodulin and the proteins involved in E–C coupling, some of which are also controlled by Ca^2+^–calmodulin, as well as many proteins responsible for the rapid generation of ATP required for fast muscle contraction, tend to be expressed at higher levels in 2X compared with 2A fibers, thus further enhancing the contractile response of 2X fibers.

Another protein highly expressed in type 2X fibers is ACTN3 (α-actinin-3). Using fiber typing for myosin ATPase in human skeletal muscle, ACTN3 was originally detected in all type 2B (now referred to as 2X) and in 50% type 2A fibers [54]. However, based on our immunofluorescence analyses using specific anti-MYH antibodies, it is likely that the 50% type 2A fibers reactive for anti-ACTN3 antibodies were in fact hybrid 2A/2X fibers containing substantial proportion of MYH1, which are numerous in human muscle and show strong staining for ACTN3 (see Fig. 8E). Proteomic analysis for the first time provides quantitative data concerning the pattern of expression of ACTN3 in human muscle fibers, showing that this α-actinin isoform is expressed at highest levels in type 2X fibers, at much lower levels (less than one third) in 2A fibers and just in traces, if any, in type 1 fibers (Table S2 and Dataset 1). Interestingly, ACTN3 is absent in many individuals due to a common ACTN3 R577X polymorphism, and the presence or absence of ACTN3 affects skeletal muscle performance, ACTN3 being associated with muscle power performance in elite sprint athletes [55]. Accordingly, type 2X fibers, which have the highest levels of ACTN3, show the maximal velocity of shortening among the different human fiber types [56].

A novel interesting finding of the present study is the demonstration that most components of the dystrophin glycoprotein complex and integrin adhesion complex are expressed at higher levels in type 2X fibers (Table S5). Both dystrophin and integrin are known to be more abundant at costameres and myotendinous junctions, namely at sites where higher tension is generated during muscle contraction. The enrichment of these proteins in 2X fibers could be related to the stronger mechanical stress that the sarcolemma of these fibers must withstand due to the powerful contractions produced by 2X fibers. Muscle power, the most relevant physiological parameter in muscle physiology, is the product of force and contraction velocity, and it is known that the maximal velocity of shortening of human type 2X fibers, which reflects the properties of myosin heavy chains, is greater in 2X than in 2A fibers (note that in early studies, human 2X fibers were called 2B) [57–59]. Interestingly, a recent study on mouse muscles reported that VCL (vinculin) and ITGA7 (integrin α7), as well as other components of the integrin complex, are more abundant in glycolytic type 2 fibers [60]. In addition, we found that also tubulin alpha-4A (TUBA4A) and beta-4B (TUB4B), the major components of muscle microtubules, as well as many microtubule-associated proteins, are enriched in 2X compared with type 1 fibers (Table S3). Microtubules bind dystrophin [61, 62] and the subsarcolemmal microtubular network, which is regularly organized in mouse type 2 but not type 1 muscle fibers [63], becomes disorganized in the dystrophin-deficient *mdx* mice but can be restored by viral delivery of mini- or micro-dystrophin [64, 65]. It is tempting to speculate that the microtubular network contributes to reinforce the sarcolemmal and subsarcolemmal systems responsive to mechanical stress. Interestingly, several proteins involved in sarcolemmal resealing after membrane damage, including DYSF (dysferlin), ANXA5 (annexin 5), and BIN1 (amphiphysin 2) [66], are also more abundant in 2X fibers (Table S5). Taken together, these findings suggest that human 2X fibers, compared with other fiber types, have a richer complement of systems able to prevent and repair membrane damage caused by fast contractile activity. Indeed, it is known that fast 2 muscle fibers, and in particular type 2X fibers, are especially susceptible to dystrophin deficiency and tend to disappear early in muscles of Duchenne muscular dystrophy patients [67, 68].

This study has a number of limitations. First, and most importantly, hundreds of muscle proteins are known to exist as distinct isoforms, which may be differentially expressed in specific fiber types, derived by alternative splicing from the same gene [69]. However, these splicing products can only be theoretically detected by our bottom–up proteomic approach when different tryptic peptides of suitable length are present in the different splice isoform. Top–down mass spectrometry has been recently used to analyze isoforms and post-translational modifications of sarcomeric proteins [70, 71]. However, this approach can only be applied to limited groups of proteins and not to the whole muscle fiber proteome. Interestingly, our database reveals fiber type–specific differences in the distribution of different heterogeneous nuclear ribonucleoproteins (hnRNPs) involved in alternative splicing, with HNRNPM enriched in type 1 fibers, and both HNRNPD and HNRNPA1 in 2X fibers. It will be of interest to determine whether these hnRNPs are involved in the generation of fiber type–specific splicing products in muscle cells.

A second limitation of all proteomic studies of skeletal muscle is the dynamic range of the proteome, whereby highly abundant sarcomeric proteins hinder the mass spectrometers from sequencing proteins present in low amount. As a consequence, many transcription factors and transcriptional coregulators could not be quantified in our dataset. One exception is TFAM (Transcription Factor A, Mitochondrial), which shows a 1 > 2A > 2X pattern of fiber type distribution like most mitochondrial proteins. A category of proteins that may be largely missing in our database is that of microproteins, often lacking tryptic peptides amenable to mass spectrometry [72] and generally absent in the protein repositories used as reference protein databases [73]. We could detect phospholamban, but no other micropeptides, such as sarcolipin (SLN) and myoregulin (MLN), which regulate the activity of the SERCA Ca^2+^ pump of the SR [74], or MOXI (MTLN), a mitochondrial micropeptide that enhances fatty acid β-oxidation [75]. Micropeptide discovery is complicated by false-positive identifications, and multiple independent approaches are required to distinguish true micropeptides, as emphasized in a recent study on the human heart [76].

## Conclusion

In conclusion, we provide here for the first time a detailed inventory of the distribution of thousands of muscle proteins in the three major fiber types present in the human skeletal muscle, based on a dataset of more than 3800 proteins, quantified by MS-based proteomic of single muscle fibers. The combination of the following three main features of the data presented here make this work a valuable resource for future investigations on the specific roles of muscle proteins and their adaptive changes in muscle physiology and pathology. (i) We present a fiber type-resolved dataset of more than 400 proteins showing statistically significant differences between fiber types and identify five major patterns of fiber type distribution in human skeletal muscle. (ii) As the starting material of our workflow are isolated fibers, our dataset is devoid of the contribution of proteins derived from non-muscle cells normally present in a total lysate. (iii) It will be a basis to detect fiber type–specific changes in the skeletal muscle. We have previously shown that glycolytic enzymes are downregulated in human type 2A but upregulated in type 1 fibers during aging [3]. More recently, fiber type–specific adaptations to exercise training were described using pools of isolated muscle fibers from freeze-dried human muscle biopsies, pointing to a future direction of fiber type–specific molecular physiology of skeletal muscle [77]. Further insight into muscle fiber molecular heterogeneity will closely follow recent developments of proteomics, which is reaching the sensitivity required for the analysis of mononucleated single cells [78]. Combined with artificial intelligence-driven image analysis and laser capture microdissection [79], ultrasensitive mass spectrometry will further advance our knowledge of skeletal muscle diversity and plasticity.

## Supplementary Information

**Additional file 1: Dataset 1.** Human muscle proteins detected by single-fiber proteomics.

**Additional file 2: Dataset 2.** Human muscle proteins showing a statistically significant difference between fiber types.

**Additional file 3: Figure S1.** Fiber type distribution of calmodulin and some Ca^2+^-calmodulin-dependent muscle proteins. **Figure S2.** Malate-aspartate shuttle: fiber type distribution of cytosolic and mitochondrial proteins. **Figure S3.** Fiber type distribution of adenylate kinase and enzymes of the purine nucleotide cycle. **Table S1.** Minor patterns of muscle proteins showing a statistically significant difference between fiber types. **Table S2.** Myofibrillar proteins, including sarcomeric cytoskeleton. **Table S3.** Cytoskeleton and cytoskeleton-associated proteins. **Table S4.** T-tubules and sarcoplasmic reticulum (SR). **Table S5.** Dystrophin and integrin complexes and membrane repair systems. **Table S6.** Glycolysis and NADH shuttles.

## Data Availability

Relevant raw proteomic data are deposited in ProteomeXchange with the accession number PXD006182.

## Abbreviations

ACO2: Aconitase 2
ACTN2: α-Actinin-2
ACTN3: α-Actinin-3
AGL: Glycogen debranching enzyme
ALDOA: Aldolase
ASPH: Junctin/junctate
ATL2: Atlastin 2
ATP2A1: SERCA 1
ATP2A2: SERCA 2
BIN1: Bridging integrator 1 (amphiphysin 2)
CACNAG1: Voltage-dependent calcium channel Ca_V_1.1 (dihydropyridine receptor, DHPR) γ1 subunit CAPZA1, CapZ α1
CASQ1: Calsequestrin 1
CASQ2: Calsequestrin 2
CS: Citrate synthase
CYCS: Cytochrome c
DLST: Dihydrolipoamide S-succinyltransferase
DNM2: Dynamin 2
ENO3: Enolase 3 (β-enolase)
FKBP1A: FK binding protein 1A, prolyl isomerase 1A
FH: Fumarate hydratase
GAPDH: Glyceraldehyde 3-phosphate dehydrogenase
GP1: Glucose-6-phosphate isomerase
GPD1: Glycerol-3-phosphate dehydrogenase 1 (cytosolic)
GPD2: Glycerol-3-phosphate dehydrogenase 2 (mitochondrial)
HRC: Histidine-rich Ca^2+^ binding protein
IDH2: Isocitrate dehydrogenase, NADPH-dependent
IDH3A: Isocitrate dehydrogenase, NADH-dependent, subunit α
IGFN1: Immunoglobulin-like and fibronectin type III domain-containing 1
JPH1: Junctophilin 1
JPH2: Junctophilin 2
JSRP1: JP-45
LDHA: Lactate dehydrogenase A
LMOD2: Leiomodin-2
LRRC39: Myomasp
MCU: Mitochondrial Ca^2+^uniporter
MDH2: Malate dehydrogenase, mitochondrial
MYBPC1: Myosin-binding protein C1
MYBPC2: Myosin-binding protein C2
MYBPH: Myosin-binding protein H
MYL1: Myosin light chain 1/3-fast (MLC1/3-fast)
MYL2: Myosin light chain 2 slow (MLC-2 slow)
MYL3: Myosin light chain 1 SLOW (MLC-1slow)
MYL6B: Myosin light chain 6B (MLC-1sa)
MYLK2: Myosin light chain kinase 2
MYLPF: MLC2-fast
MYOM2: Myomesin 2
MYOM3: Myomesin 3
MYOZ2: Myozenin 2
MYOZ3: Myozenin 3
OGDH: Oxoglutarate dehydrogenase
PFKM: Phosphofructokinase, muscle isoform
PGAM2: Phosphoglycerate mutase 2
PGK1: Phosphoglycerate kinase 1
PGM1: Phosphoglucomutase 1
PKM: Pyruvate kinase, muscle isoform
PLN: Phospholamban
PYGM: Glycogen phosphorylase
RTN2: Reticulon 2
RTN4: Reticulon 4
RYR1: Ryanodine receptor 1
SDHA: Succinate dehydrogenase subunit A
SDHB: Succinate dehydrogenase subunit B
SRL: Sarcalumenin
STAC3: SH3 and cysteine-rich domain-containing protein 3
SUCLA2: Succinate-CoA ligase ADP-forming subunit β
SUCLG1: Succinate-CoA ligase GDP/ADP-forming subunit α
SYPL2: Mg29 (mitsugumin 29)
TMEM38A: TRIC-A (trimeric intracellular cation channel A)
TMEM38B: TRIC-B
TNNC1: Slow troponin C
TNNC2: Fast troponin C
TNNC1: Slow troponin C
TNNC2: Fast troponin C
TNNI1: Slow troponin I
TNNI2: Fast troponin I
TNNT1: Slow troponin T
TNNT2: Fast troponin T
TPI1: Triosephosphate isomerase 1
TPM1: α-Tropomyosin
TPM3: γ-Tropomyosin
TRDN: Triadin
