# Supplementary material for: Protein profile of fiber types in human skeletal muscle: a single-fiber proteomics study
Source: Skelet Muscle. 2021 Nov 2;11:24. doi: 10.1186/s13395-021-00279-0 (PMC8561870; doi:10.1186/s13395-021-00279-0)
Supplement: Supplementary file 3 — Additional file 3: Figure S1. Fiber type distribution of calmodulin and some Ca2+-calmodulin-dependent muscle proteins. Figure S2. Malate-aspartate shuttle: fiber type distribution of cytosolic and mitochondrial proteins. Figure S3. Fiber type distribution of adenylate kinase and enzymes of the purine nucleotide cycle. Table S1. Minor patterns of muscle proteins showing a statistically significant difference between fiber types. Table S2. Myofibrillar proteins, including sarcomeric cytoskeleton. Table S3. Cytoskeleton and cytoskeleton-associated proteins. Table S4. T-tubules and sarcoplasmic reticulum (SR). Table S5. Dystrophin and integrin complexes and membrane repair systems. Table S6. Glycolysis and NADH shuttles. [file 13395_2021_279_MOESM3_ESM.docx]

**ADDITIONAL FILE 3**

**Protein profile of fiber types in human skeletal muscle. A single-fiber proteomics study**

Marta Murgia, Leonardo Nogara, Martina Baraldo, Carlo Reggiani, Matthias Mann, Stefano Schiaffino

This PDF file includes:

Supplemental Figures S1-S3 with legends

Supplemental Tables S1-S6

(*Supplemental datasets 1 and 2 are found in Additional file 1 and 2, respectively*)

**-----------**

**List of supplemental figures and tables**

Figure S1. Fiber type distribution of calmodulin and some Ca^2+^-calmodulin-dependent muscle proteins

Figure S2. Malate-aspartate shuttle: fiber type distribution of cytosolic and mitochondrial proteins

Figure S3. Fiber type distribution of adenylate kinase and enzymes of the purine nucleotide cycle

Table S1. Minor patterns of muscle proteins showing a statistically significant difference between fiber types

Table S2. Myofibrillar proteins, including sarcomeric cytoskeleton

Table S3. Cytoskeleton and cytoskeleton-associated proteins

Table S4. T-tubules and sarcoplasmic reticulum (SR)

Table S5. Dystrophin and integrin complexes and membrane repair systems

Table S6. Glycolysis and shuttles

**-----------**


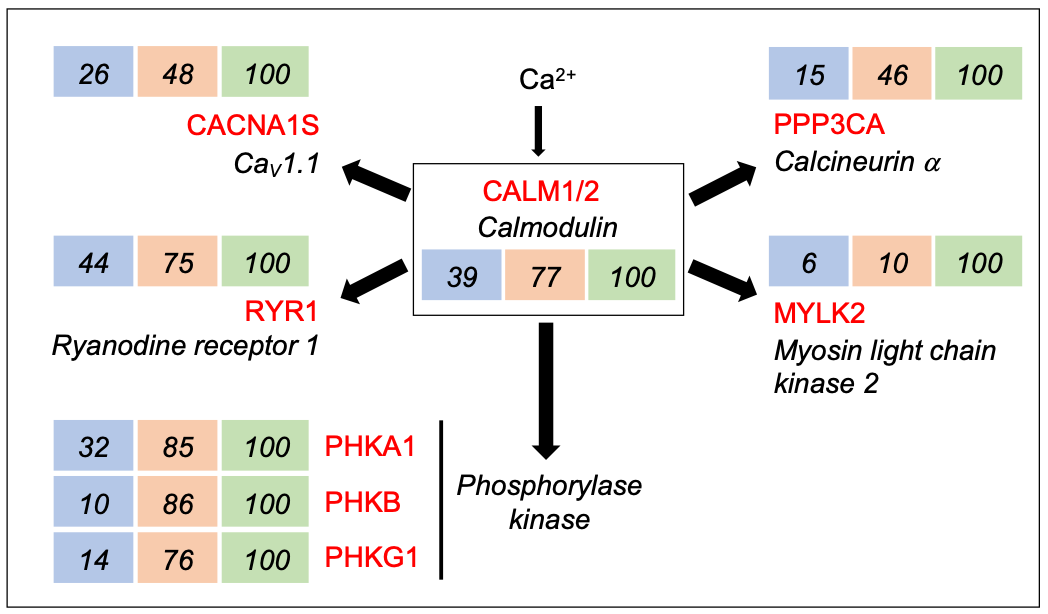


**Figure S1.** **Fiber type distribution of calmodulin and some Ca^2+^-calmodulin-dependent muscle proteins**. Values are expressed as per cent of the maximal value. CACNA1S, voltage-dependent calcium channel Ca_V_1.1 (dihydropyridine receptor) α1S subunit; PHKA1, PHKB, PHKG1: glycogen phosphorylase kinase subunits α1, β and γ1.

**
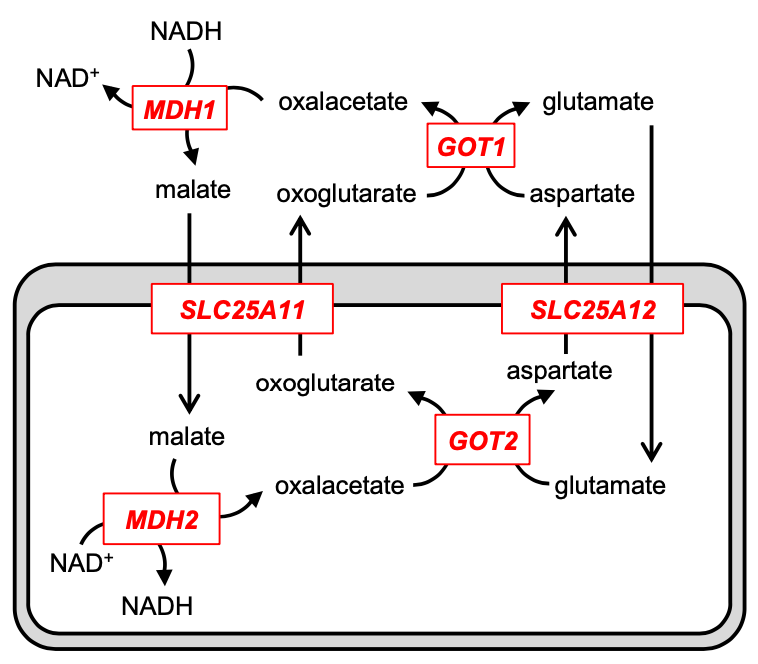
**

**
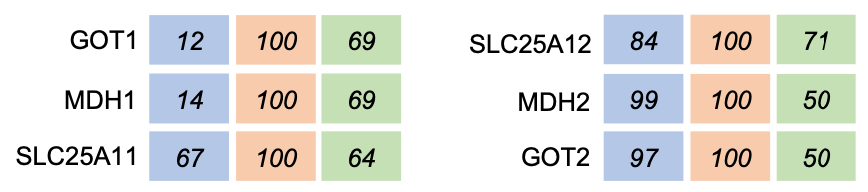
**

**Figure S2. Malate-aspartate shuttle: fiber type distribution of cytosolic and mitochondrial proteins.** A scheme of the shuttle is shown in the upper panel and a list of the constituent enzymes with their relative abundance in the different fiber types in the lower panel. Values are expressed as per cent of the maximal value. GOT1, aspartate amino-transferase, cytosolic; GOT2, aspartate amino-transferase, mitochondrial; MDH1, malate dehydrogenase, cytosolic; MDH2, malate dehydrogenase, mitochondrial; SLC25A11, oxoglutarate-malate carrier; SLC25A12, aspartate-glutamate carrier.

**
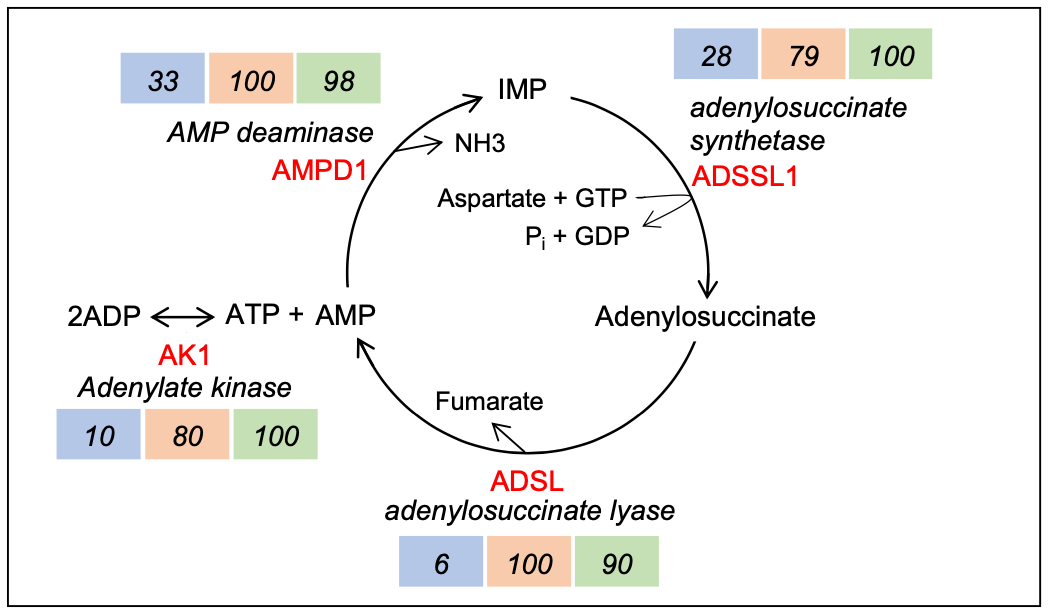
**

**Figure S3. Fiber type distribution of adenylate kinase and enzymes of the purine nucleotide cycle**. Values are expressed as per cent of the maximal value.

**Table S1. Minor patterns of muscle proteins showing a statistically significant difference between fiber types**

| *Pattern* | *Fiber type difference ^1^* | *Threshold* | *No. of proteins* |
| --- | --- | --- | --- |
|  |  |  |  |
| Type 2A-specific | 2a>1;2a>2x | >3 times | 2 |
| Type 1/2A-specific | 1>2x; 2a>2x | >3 times | 4 |
| Type 1-specific | 1>2a;1>2x | <3 times | 5 |
| Type 2A-specific | 2a>1;2a>2x | <3 times | 3 |
| Type 2X-specific | 2x>1;2x>2a | <3 times | 7 |
| Type 1/2A-specific | 1>2x; 2a>2x | <3 times | 1 |
| Type 2A/2X-specific | 2x>1;2a>1 | <3 times | 12 |
|  | 1>2a | - | 17 |
|  | 2a>1 | - | 16 |
|  | 2a>2x | - | 1 |
|  | 2x>2a |  | 1 |
|  | 1>2a, 2x>2a | - | 12 |
|  | 1>2a;2x>2a;2x>1 | - | 1 |
| *Total* |  |  | *82* |

*^1^ p* < 0,05

**Table S2. Myofibrillar proteins, including sarcomeric cytoskeleton ^1^**

| **Gene name** | **Protein name** | **Fiber type expression**  (median, absolute values) | | | **Significant fiber type difference**^2^ | **Fiber type expression**  (median, % of max value) | | |
| --- | --- | --- | --- | --- | --- | --- | --- | --- |
|  |  | **1** | **2A** | **2X** |  | **1** | **2A** | **2X** |
| **Predominant in slow fibers** | |  |  |  |  |  |  |  |
| MYL2 | MLC-slow | 66804,4 | 120,8 | 616,4 | 1>2a;1>2x | 100 | .2 | .9 |
| MYL3 | MLC1-slow | 62341,8 | 5355,4 | 6341,0 | 1>2a;1>2x | 100 | 9 | 10 |
| MYL6B | MLC-1sa | 2338,8 | 147,8 | 169,1 | 1>2a;1>2x | 100 | 6 | 7 |
| TPM3 | γ-tropomyosin | 59095,1 | 25138,9 | 24424,1 | 1>2a;1>2x | 100 | 43 | 41 |
| TNNC1 | Slow troponin C | 61876,7 | 192,2 | 170,0 | 1>2a;1>2x | 100 | .3 | .3 |
| TNNI1 | Slow troponin I | 51247,8 | 411,6 | 369,5 | 1>2a;1>2x | 100 | .8 | .7 |
| TNNT1 | Slow troponin T | 47093,4 | 19865,0 | 18412,2 | 1>2a;1>2x | 100 | 42 | 39 |
| MYBPC1 | Myosin binding protein C1 | 11475,8 | 9280,4 | 7269,5 | 1>2a;1>2x;2a>2x | 100 | 81 | 63 |
| ACTN2 | α-actinin-2 | 15565,3 | 12167,5 | 8652,2 | 1>2a;1>2x | 100 | 78 | 56 |
| MYOZ2 | Myozenin 2 (FATZ 2, calsarcin 1) | 1592,1 | 36,4 | 27,3 | 1>2a;1>2x | 100 | 2 | 2 |
| MYOM3 | Myomesin 3 | 1939,8 | 481,0 | 169,8 | 1>2a;1>2x;2a>2x | 100 | 25 | 9 |
| LRRC39 | Myomasp | 49.0 | 16.0 | 4.0 | 1>2a;1>2x;2a>2x | 100 | 33 | 8 |
| LMOD2 | Leiomodin 2 | 34,0 | 9,7 | 9,0 | 1>2a;1>2x | 100 | 29 | 26 |
| KLHL40 | Kelch-like 40 | 354,9 | 231,5 | 150,0 | 1>2x:2a>2x | 100 | 65 | 42 |
| **Predominant in fast fibers** | |  |  |  |  |  |  |  |
| MYL1 | MLC-1/3 fast | 7974,7 | 33756,4 | 32551,2 | 2x>1;2a>1 | 24 | 100 | 96 |
| MYLPF | MLC2-fast | 1157,5 | 66901,1 | 68573,7 | 2x>1;2a>1 | 2 | 98 | 100 |
| TPM1 | α-tropomyosin | 812,9 | 34785,0 | 25658,4 | 2x>1;2a>1 | 2 | 100 | 74 |
| TNNC2 | Fast troponin C | 953,2 | 81500,4 | 89661,7 | 2x>1;2a>1 | 1 | 91 | 100 |
| TNNI2 | Fast troponin I | 119,6 | 35012,8 | 41049,8 | 2x>1;2a>1 | .3 | 85 | 100 |
| TNNT3 | Fast troponin T | 213,9 | 34155,5 | 33501,1 | 2x>1;2a>1 | 6 | 100 | 98 |
| MYBPC2 | Myosin binding protein C2 | 24,6 | 4284,3 | 12766,5 | 2x>1;2a>1; 2x>2a | .2 | 34 | 100 |
| ACTN3 | α-actinin-3 | 74,3 | 2403,1 | 8461,3 | 2x>1;2a>1; 2x>2a | .9 | 28 | 100 |
| MYOZ3 | Myozenin 3 (FATZ3, calsarcin 3) | 773,7 | 1395,0 | 1709,2 | 2x>1;2a>1 | 45 | 82 | 100 |
| CAPZA1 | CapZ α1 | 10,4 | 10,0 | 61,3 | 2x>1;2x>2a | 17 | 16 | 100 |
| **No significant fiber type difference** | |  |  |  |  |  |  |  |
| TTN | Titin | 3878,7 | 4358,1 | 4381,8 | ns | 89 | 99 | 100 |
| NEB | Nebulin | 2755,9 | 2815,9 | 2812,0 | ns | 98 | 100 | 100 |
| OBSCN | Obscurin | 368,4 | 304,9 | 232,8 | ns | 100 | 83 | 63 |
| TPM2 | β-tropomyosin | 30031,9 | 29861,8 | 27517,3 | ns | 100 | 99 | 92 |
| MYBPH | Myosin binding protein H | 17,3 | 28,9 | 58,1 | ns | 30 | 50 | 100 |
| MYOZ1 | Myozenin 1 (FATZ, calsarcin 2) | 4585,5 | 5648,7 | 5523,3 | ns | 81 | 100 | 98 |
| MYOM1 | Myomesin 1 | 2496,4 | 2446,4 | 2178,0 | ns | 100 | 98 | 87 |
| MYOM2 | Myomesin 2 (M-protein) | 3000,4 | 5176,3 | 5557,0 | ns | 54 | 93 | 100 |
| MYOT | Myotilin | 1900,8 | 1211,9 | 801,1 | ns | 100 | 64 | 42 |
| CAPZB | CapZ β | 797,4 | 931,2 | 942,2 | ns | 85 | 99 | 100 |
| CAPZA2 | CapZ α2 | 304,8 | 325,2 | 364,5 | ns | 84 | 89 | 100 |
| TCAP | Tcap, telethonin | 916,9 | 938,6 | 972,6 | ns | 94 | 97 | 100 |
| SYNPO2 | Myopodin, Synaptopodin-2 | 437,8 | 513,1 | 449,5 | ns | 73 | 35 | 100 |
| MYPN | Myopalladin | 89,1 | 88,9 | 97,5 | ns | 91 | 91 | 100 |
| TMOD4 | Tropomodulin 4 | 1412,3 | 1373,5 | 1474,1 | ns | 96 | 93 | 100 |
| CFL2 | Cofilin 2 | 497,9 | 952,8 | 683,4 | ns | 52 | 100 | 72 |
| LMOD3 | Leiomodin 3 | 133,9 | 97,9 | 125,0 | ns | 100 | 73 | 93 |
| KLHL41 | Kelch-like 41 | 3417,6 | 2979,7 | 2567,2 | ns | 100 | 87 | 75 |

^1^ MYHs are not included.

^2^ *p* < 0,05

**Table S3. Cytoskeleton and cytoskeleton-associated proteins**

| **Gene name** | **Protein name** | **Fiber type expression**  (median, absolute values) | | | **Significant fiber type difference**^1^ | **Fiber type expression**  (median, % of max value) | | |
| --- | --- | --- | --- | --- | --- | --- | --- | --- |
|  |  | **1** | **2A** | **2X** |  | **1** | **2A** | **2X** |
| **Intermediate filaments** | |  |  |  |  |  |  |  |
| DES | Desmin | 6504,3 | 4521,1 | 3542,9 | 1>2x;2a>2x | 100 | 70 | 54 |
| FLNC | Filamin C | 3610,4 | 4351,0 | 2858,3 | ns | 83 | 100 | 66 |
| SYNM | Synemin | 666,0 | 642,3 | 567,3 | ns | 100 | 96 | 30 |
| PLEC | Plectin | 1017,1 | 783,4 | 618,9 | ns | 100 | 77 | 61 |
| SYNC | Syncoilin | 11,3 | 13,1 | 22,3 | ns | 50 | 59 | 100 |
| NES | Nestin | 10,3 | 17,6 | 6,5 | ns | 59 | 100 | 37 |
| **LIM domain containing proteins** | |  |  |  |  |  |  |  |
| PDLIM1 | PDLIM1, CLP36 | 387,9 | 13,4 | 22,1 | 1>2x;1>2a | 100 | 3 | 6 |
| PDLIM3 | PDLIM3, ALP | 329,0 | 739,7 | 1215,5 | ns | 27 | 61 | 100 |
| PDLIM7 | PDLIM7, Enigma | 123,1 | 463,6 | 1503,2 | 2a>1;2x>1;2x>2a | 8 | 31 | 100 |
| LDB3 | PDLIM6, ZASP, Cypher, Oracle | 2762,2 | 2572,0 | 2116,8 | ns | 78 | 91 | 100 |
| FHL1 | Four and a half LIM domains 1 | 2409,5 | 9905,5 | 6845,5 | ns | 24 | 100 | 69 |
| FHL3 | Four and a half LIM domains 3 | 1514,5 | 1190,7 | 1704,1 | ns | 89 | 70 | 100 |
| CSRP3 | MLP (muscle LIM protein) | 375,3 | 744,8 | 103,2 | 2a>2x | 50 | 100 | 14 |
| LMCD1 | LIM and cysteine rich domain 1 | 145,6 | 188,1 | 62,6 | ns | 77 | 100 | 33 |
| **Other cytoskeleton-associated proteins** | |  |  |  |  |  |  |  |
| ANKRD2 | ANKRD2, Arpp | 201,7 | 259,0 | 33,5 | 1>2x;2a>2x | 78 | 100 | 13 |
| ANKRD35 | ANKRD35 | 8,3 | 219,4 | 198,2 | 2x>1;2a>1 | 4 | 100 | 90 |
| FHOD1 | Formin-1 | 20,1 | 93,7 | 77,3 | 2x>1;2a>1 | 21 | 100 | 82 |
| COBL | Cordon-Bleu WH2 Repeat Protein | 5.0 | 8.2 | 21.8 | 2x>1;2a>1 | 23 | 38 | 100 |
| IGFN1 | Ig-like & Fn Type III Domain 1 | 3.2 | 27.6 | 8.9 | ns | 12 | 100 | 32 |
| NRAP | Nebulin-related anchoring protein | 194,4 | 199,5 | 149,1 | ns | 97 | 100 | 75 |
| XIRP1 | Xin | 136,5 | 105,2 | 53,5 | ns | 100 | 77 | 39 |
| XIRP2 | Xin2, beta-Xin | 1,7 | 18,3 | 42,9 | ns | 4 | 43 | 100 |
| PGM5 | Aciculin | 66,8 | 11,6 | 5,5 | 1>2x;1>2a | 100 | 17 | 8 |
| CMYA5 | Myospryn | 41,9 | 49,7 | 52,5 | ns | 80 | 95 | 100 |
| SMTNL1 | Smoothelin-like 1 | 29,2 | 84,6 | 37,1 | ns | 35 | 100 | 44 |
| SMTNL2 | Smoothelin-like 2 | 42,9 | 57,2 | 123,8 | 2x>1;2x>2a | 35 | 46 | 100 |
| **Microtubules and associated proteins** | |  |  |  |  |  |  |  |
| TUBA1A | Tubulin alpha 1A | 147,0 | 240,4 | 385,3 | ns | 38 | 62 | 100 |
| TUBA1B | Tubulin alpha 1B | 10,3 | 26,1 | 43,7 | ns | 24 | 60 | 100 |
| TUBA4A | Tubulin alpha 4A | 188,9 | 361,3 | 533,5 | 2x>1 | 35 | 68 | 100 |
| TUBA8 | Tubulin alpha 8 | 23,0 | 25,9 | 27,1 | ns | 85 | 96 | 100 |
| TUBB | Tubulin beta | 60,3 | 71,4 | 89,3 | ns | 68 | 80 | 100 |
| TUBB2B | Tubulin beta 2B | 59,5 | 57,5 | 66,4 | ns | 90 | 87 | 100 |
| TUBB4B | Tubulin beta 4B | 88,8 | 321,0 | 691,6 | 2x>1;2a>1 | 13 | 46 | 100 |
| MAP1A | MT- associated protein 1A | 1,5 | 4,6 | 9,1 | ns | 16 | 51 | 100 |
| MAP1LC3B | MAP1LC3B | 13,7 | 48,3 | 58,0 | 2x>1;2a>1 | 24 | 83 | 100 |
| MAP4 | MAP4 | 85,9 | 94,0 | 116,3 | ns | 74 | 81 | 100 |
| MAPT | MAP Tau | 11,1 | 48,1 | 44,3 | ns |  |  |  |
| MAPRE2 | MAP RP/EB 2 | 11,3 | 14,7 | 39,9 | ns | 28 | 37 | 100 |
| CLIP1 | Cytoplasmic linker protein 1 | 2.8 | 6.5 | 6.4 | ns | 44 | 100 | 99 |
| CNP | Cyclic-nucleotide 3'-phosphodiesterase | 5,8 | 4,2 | 34,8 | 2x>1;2x>2a | 17 | 12 | 100 |
| FKBP3 | FK506 binding protein 3, FKBP25 | 55,2 | 115,5 | 156,8 | ns | 35 | 74 | 100 |
| TBCA | Tubulin Folding Cofactor A | 52,9 | 89,5 | 183,7 | ns | 29 | 49 | 100 |
| HOOK3 | Hook microtubule tethering protein 3 | 7,4 | 6,5 | 15,8 | ns | 47 | 41 | 100 |

^1^ *p* < 0,05

**Table S4. T-tubules and sarcoplasmic reticulum (SR)**

| **Gene name** | **Protein name** | **Fiber type expression**  (median, absolute values) | | | **Significant fiber type difference**^1^ | **Fiber type expression**  (median, % of max value) | | |
| --- | --- | --- | --- | --- | --- | --- | --- | --- |
|  |  | **1** | **2A** | **2X** |  | **1** | **2A** | **2X** |
| **T-tubules** |  |  |  |  |  |  |  |  |
| CACNA1S | Cav α1s (Cav1.1), dihydro-pyridine receptor (DHPR) | 53,9 | 103,1 | 96,8 | ns | 52 | 100 | 94 |
| CACNA2D1 | Cav α2/δ1 | 96,9 | 179,6 | 372,6 | 2x>1;2x>2a;2a>1 | 26 | 48 | 100 |
| CACNB1 | Cav β1 | 89,9 | 185,8 | 243,9 | ns | 37 | 76 | 100 |
| CACNG1 | Cav γ1 | 65,9 | 102,7 | 254,2 | 2x>1;2x>2a | 26 | 40 | 100 |
| STAC3 | SH3 and cysteine-rich protein 3 | 48,2 | 103,3 | 142,4 | 2x>1;2x>2a;2a>1 | 34 | 73 | 100 |
| SYPL2 | Synaptophysin-like protein 2, MG29 | 2011,9 | 1988,8 | 4373,6 | 2x>1;2x>2a | 46 | 45 | 100 |
| BIN1 | BIN1, amphiphysin 2 | 291,7 | 597,1 | 1090,3 | 2x>1;2x>2a | 27 | 55 | 100 |
| DNM2 | Dynamin 2 | 5,3 | 10,5 | 17,0 | ns | 31 | 62 | 100 |
| **Sarcoplasmic reticulum (SR)** | |  |  |  |  |  |  |  |
| RYR1 | Ryanodine receptor 1 | 201,2 | 344,6 | 460,1 | 2x>1;2a>1 | 44 | 75 | 100 |
| TRDN | Triadin | 623,5 | 1631,8 | 1832,9 | 2x>1;2a>1 | 34 | 89 | 100 |
| JPH1 | Junctophilin-1 | 171,3 | 333,8 | 412,5 | 2x>1;2a>1 | 42 | 81 | 100 |
| JPH2 | Junctophilin-2 | 153,4 | 274,7 | 287,2 | 2x>1;2a>1 | 53 | 96 | 100 |
| ASPH | Junctin/Junctate | 2411,4 | 4116,0 | 7212,0 | 2x>1;2x>2a | 33 | 57 | 100 |
| JSRP1 | JP-45 | 209,9 | 328,9 | 632,7 | 2x>1;2x>2a;2a>1 | 33 | 52 | 100 |
| ATP2A1 | SERCA1 | 148,4 | 9106,6 | 13760,3 | 2x>1;2a>1 | 1 | 66 | 100 |
| ATP2A2 | SERCA2 | 10287,6 | 4911,3 | 7304,7 | ns | 100 | 48 | 71 |
| FKBP1A | FK506 binding protein 1A, calstabin | 348,5 | 734,8 | 1060,0 | 2x>1;2x>2a | 33 | 69 | 100 |
| PLN | Phospholamban | 1867,5 | 558,6 | 193,6 | 1>2x;1>2a | 100 | 30 | 10 |
| CASQ1 | Calsequestrin 1 | 19347,2 | 25884,9 | 37901,9 | 2x>1;2x>2a;2a>1 | 51 | 68 | 100 |
| CASQ2 | Calsequestrin 2 | 3133,4 | 382,2 | 116,7 | 1>2x;1>2A;2a>2x | 100 | 12 | 4 |
| SRL | Sarcalumenin | 2104,7 | 1819,0 | 2015,8 | ns | 100 | 86 | 96 |
| HRC | Histidine Rich Ca^2+^ Binding Protein | 298,6 | 267,7 | 240,2 | ns | 100 | 90 | 80 |
| TMEM38A | TRIC-A, SRP27 | 189,4 | 203,3 | 218,4 | ns | 87 | 93 | 100 |
| TMEM38B | TRIC-B | 44,8 | 52,3 | 97,8 | ns | 46 | 53 | 100 |
| MLEC | Malectin | 29.6 | 33.6 | 90.0 | 2x>1;2a>1 | 33 | 37 | 100.0 |
| CALR | Calreticulin | 186.9 | 140.2 | 140.6 | ns | 76 | 94 | 100 |
| CANX | Calnexin | 180.5 | 223.0 | 237.5 | ns | 100 | 75 | 75 |
| RTN4 | Reticulon 4 | 461,4 | 493,6 | 849,4 | 2x>1;2x>2a | 54 | 58 | 100 |
| RTN2 | Reticulon 2 | 777,3 | 784,9 | 1383,3 | 2x>1;2x>2a | 56 | 57 | 100 |
| ATL2 | Atlastin 2 | 13,4 | 39,0 | 65,0 | 2x>1;2a>1 | 21 | 60 | 100 |
| REEP5 | DP1 | 771,3 | 734,1 | 1142,8 | ns | 67 | 64 | 100 |

^1^ *p* < 0,05

**Table S5. Dystrophin and integrin complexes and membrane repair systems**

| **Gene name** | **Protein name** | **Fiber type expression**  (median, absolute values) | | | **Significant fiber type difference**^1^ | **Fiber type expression**  (median, % of max value) | | |
| --- | --- | --- | --- | --- | --- | --- | --- | --- |
|  |  | **1** | **2A** | **2X** |  | **1** | **2A** | **2X** |
| **Dystrophin complex** | |  |  |  |  |  |  |  |
| DMD | Dystrophin | 156,5 | 197,2 | 231,1 | ns | 68 | 85 | 100 |
| DAG1 | Dystroglican | 68,0 | 62,7 | 209,8 | ns | 32 | 30 | 100 |
| SGCA | Sarcoglycan α | 151,4 | 177,4 | 209,1 | ns | 72 | 85 | 100 |
| SGCB | Sarcoglycan β | 61,8 | 49,7 | 69,0 | ns | 90 | 72 | 100 |
| SGCD | Sarcoglycan δ | 141,0 | 120,8 | 225,3 | 2x>1;2x>2a | 63 | 54 | 100 |
| SGCG | Sarcoglycan γ | 468,2 | 210,6 | 423,2 | ns | 100 | 45 | 90 |
| SSPN | Sarcospan | 23,4 | 78,0 | 143,4 | 2x>1;2x>2a | 16 | 55 | 100 |
| DTNA | Dystrobrevin α | 53,6 | 66,8 | 80,1 | ns | 67 | 83 | 100 |
| SNTB1 | β1-syntrophin | 41,8 | 1469,5 | 698,7 | 2x>1;2a>1 | 3 | 100 | 48 |
| SNTA1 | α1-syntrophin | 81,3 | 98,5 | 122,0 | ns | 67 | 81 | 100 |
| **Integrin complex** | |  |  |  |  |  |  |  |
| ITGA1 | Integrin α1 | 3.1 | 4.1 | 22.4 | ns | 14 | 18 | 100 |
| ITGA7 | Integrin α7 | 8,4 | 21,4 | 27,7 | ns | 30 | 77 | 100 |
| ITGB1 | Integrin β1 | 5,5 | 11,3 | 29,5 | 2x>1;2x>2a | 19 | 38 | 100 |
| TLN1 | Talin 1 | 10,3 | 15,6 | 21,5 | ns | 48 | 72 | 100 |
| TLN2 | Talin 2 | 15,1 | 15,6 | 14,8 | ns | 97 | 100 | 95 |
| TNS1 | Tensin-1 | 11,5 | 10,8 | 18,0 | 2x>1;2x>2a | 64 | 60 | 100 |
| VCL | Vinculin | 88,0 | 135,1 | 137,9 | ns | 64 | 98 | 100 |
| PARVB | Parvin B | 7,9 | 7,1 | 25,1 | ns | 32 | 28 | 100 |
| SORBS2 | Sorbin And SH3 Domain Containing 2 (ArgBP2) | 13.3 | 20.9 | 99.7 | ns | 13 | 21 | 100 |
| **Membrane repair systems** | |  |  |  |  |  |  |  |
| DYSF | Dysferlin | 117,3 | 166,3 | 211,8 | 2x>1;2x>2a | 55 | 79 | 100 |
| ANXA2 | Annexin 2 | 122,5 | 109,6 | 167,8 | ns | 73 | 65 | 100 |
| ANXA5 | Annexin 5 | 8,3 | 35,5 | 72,2 | 2x>1;2a>1 | 12 | 49 | 100 |
| ANXA6 | Annexin 6 | 46,9 | 195,7 | 225,0 | ns | 21 | 87 | 100 |
| EHD1 | Eps15 homology domain-containing 1 | 16,8 | 17,1 | 26,3 | ns | 64 | 65 | 100 |
| TRIM72 | MG53 | 1510,8 | 1555,0 | 1330,8 | ns | 97 | 100 | 86 |
| PTRF | CAVIN-1 (Caveolae Associated Protein 1) | 85,5 | 96,2 | 196,9 | ns | 43 | 49 | 100 |
| BIN1 | Amphiphysin 2 | 291,7 | 597,1 | 1090,3 | 2x>1;2x>2a | 57 | 55 | 100 |
| LGALS1 | Galectin-1 | 202,6 | 671,0 | 1147,3 | 2x>1;2a>1 | 18 | 58 | 100 |

^1^ *p* < 0,05

**Table S6. Glycolysis and NADH shuttles**

| **Gene name** | **Protein name** | **Fiber type expression**  (median, absolute values) | | | **Significant fiber type difference**^1^ | **Fiber type expression**  (median, % of max value) | | |
| --- | --- | --- | --- | --- | --- | --- | --- | --- |
|  |  | **1** | **2A** | **2X** |  | **1** | **2A** | **2X** |
| **Glycolysis** |  |  |  |  |  |  |  |  |
| PGM1 | Phosphoglucomutase 1 | 78,5 | 1022,9 | 1424,1 | 2x>1;2a>1 | 6 | 72 | 100 |
| GPI | Glucose-6-P isomerase | 53,5 | 603,3 | 1064,6 | 2x>1;2a>1 | 5 | 57 | 100 |
| PFKM | Phosphofructokinase, muscle isoform | 266,0 | 1709,4 | 1752,7 | 2x>1;2a>1 | 15 | 98 | 100 |
| ALDOA | Aldolase | 1483,8 | 21545,9 | 23892,0 | 2x>1;2a>1 | 6 | 90 | 100 |
| TPI1 | Triosephosphate isomerase 1 | 362,8 | 1916,7 | 1757,4 | 2x>1;2a>1 | 19 | 100 | 92 |
| GAPDH | Glyceraldehyde 3-P dehydrogenase | 1659,9 | 14894,1 | 23238,1 | 2x>1;2a>1 | 7 | 64 | 100 |
| PGK1 | Phosphoglycerate kinase 1 | 202,8 | 2240,8 | 2106,0 | 2x>1;2a>1 | 9 | 100 | 94 |
| PGAM2 | Phosphoglycerate mutase | 291,3 | 2683,9 | 2775,2 | ns | 11 | 97 | 100 |
| ENO3 | Enolase 3 (β-enolase) | 2388,0 | 12082,9 | 13592,2 | 2x>1;2a>1 | 18 | 89 | 100 |
| PKM | Pyruvate kinase, muscle isoform | 488,9 | 5197,9 | 8660,6 | 2x>1;2a>1 | 6 | 60 | 100 |
| LDHA | Lactate dehydrogenase A | 262,5 | 3970,7 | 5787,1 | 2x>1;2a>1 | 5 | 69 | 100 |
| **Glycogenolysis** |  |  |  |  |  |  |  |  |
| PYGM | Glycogen phosphorylase | 2372,2 | 7842,4 | 10155,4 | 2x>1;2a>1 | 5 | 57 | 100 |
| PHKA1 | Glycogen phosphorylase kinase α1 | 63,0 | 169,0 | 198,3 | ns | 32 | 85 | 100 |
| PHKB | Glycogen phosphorylase kinase β | 24,1 | 212,3 | 248,4 | 2x>1;2a>1 | 10 | 86 | 100 |
| PHKG1 | Glycogen phosphorylase kinase γ1 | 42,5 | 230,2 | 304,2 | 2x>1;2a>1 | 14 | 76 | 100 |
| AGL | Glycogen debranching enzyme | 482,4 | 1055,3 | 1247,4 | 2x>1;2a>1 | 39 | 85 | 100 |
| **Glycerol-phosphate shuttle** | |  |  |  |  |  |  |  |
| GPD1 | Glycerol-P dehydrogenase 1 | 65,7 | 326,1 | 780,9 | 2x>1;2a>1 | 8 | 42 | 100 |
| GPD2 | Glycerol-P dehydrogenase 2 | 54,9 | 167,6 | 298,9 | 2x>1;2a>1;2x>2a | 18 | 56 | 100 |
| **Malate-aspartate shuttle** | |  |  |  |  |  |  |  |
| MDH1 | Malate dehydrogenase, cytosolic | 82,4 | 604,4 | 415,2 | ns | 14 | 100 | 69 |
| GOT1 | Aspartate amino-transferase, cytosolic | 101,6 | 830,4 | 574,6 | ns | 12 | 100 | 69 |
| SLC25A11 | Oxoglutarate-malate carrier | 722,7 | 1086,6 | 700,6 | ns | 67 | 100 | 64 |
| SLC25A12 | Aspartate-glutamate carrier (Aralar) | 789,7 | 943,3 | 670,7 | ns | 84 | 100 | 71 |
| SLC25A13 | Aspartate-glutamate carrier (Citrin) | 52,4 | 91,0 | 66,8 | ns | 58 | 100 | 73 |
| MDH2 | Malate dehydrogenase, mitochondrial | 9443,6 | 9558,5 | 4746,8 | ns | 99 | 100 | 50 |
| GOT2 | Aspartate amino-transferase, mitochondrial | 3233,4 | 3330,9 | 1663,9 | ns | 97 | 100 | 50 |

^1^ *p* < 0,05
